# BCAT2 binding to PCBP1 regulates the PI3K/AKT signaling pathway to inhibit autophagy-related apoptosis and ferroptosis in prostate cancer

**DOI:** 10.1038/s41419-025-07559-3

**Published:** 2025-04-24

**Authors:** Wangli Mei, Mengyu Wei, Chaozhi Tang, Weiyi Li, Bowen Ye, Shiyong Xin, Weiguo Ma, Lin Ye

**Affiliations:** 1 Department of Urology, Shanghai East Hospital, School of Medicine, Tongji University, Shanghai, 200120 China; 2 Urologic Cancer Institute, School of Medicine, Tongji University, Shanghai, 200072 China; 3 Department of Urology, First Affiliated Hospital, and College of Clinical Medicine of Henan University of Science and Technology, Luoyang, 471003 China; 4 Department of Urology, Tongxin People’s Hospital, Ningxia, 751300 China

**Keywords:** Cancer metabolism, Prostate cancer, Tumour biomarkers

## Abstract

Prostate cancer (PCa) has emerged as a predominant cause of cancer-related mortality among men globally. The mechanisms of branched-chain amino acids (BCAAs) contributing to the development of PCa remain inadequately elucidated. The objective of this study was to examine the involvement of BCAAs and BCAT2 in tumorigenesis. BCAAs exhibited elevated expression levels in PCa tissues and cells. Among the critical enzymes involved in the BCAA metabolic pathway, only BCAT2 demonstrated significant expression in PCa and was closely associated with tumor progression and patient prognosis. RNA sequencing along with related functional experiments indicated that BCAT2 can inhibit autophagy, autophagy-related apoptosis, and ferroptosis in PCa. Furthermore, the results of co-immunoprecipitation, mass spectrometry, and other methodologies established that PCBP1, as a downstream protein interacting with BCAT2, co-regulates the PI3K/AKT pathway, thereby influencing progression of PCa. Moreover, BCAT2 interacted with PCBP1 at Leucine 239 to collaboratively regulate the PI3K/AKT signaling pathway, which is crucial for the initiation and progression of PCa. Targeting BCAT2 may represent a promising therapeutic strategy to prevent proliferation of PCa.

## Introduction

Prostate cancer (PCa) represents a significant global health concern, posing a serious threat to men’s health, with the highest incidence and mortality rates among male malignant neoplasms [1]. Surgical intervention and localized radiotherapy are effective treatments for early-stage PCa. In contrast, androgen deprivation therapy provides only temporary efficacy for locally advanced and metastatic PCa, as the majority of cases ultimately progress to a castration-resistant state [2]. Consequently, further investigations are imperative to clarify the pathogenesis and molecular mechanisms underlying PCa, and to identify biomarkers for early detection and prediction of treatment responses and prognoses, as well as exploration of new therapeutic targets.

Branched-chain amino acids (BCAAs), including leucine (Leu), isoleucine (Ile), and valine (Val), are essential for normal human growth and development [3, 4], but have also been linked to various diseases, such as diabetes [5, 6], pancreatic cancer [7, 8], and bladder cancer [9], among others [10–12]. BCAA levels are primarily regulated by the branched-chain aminotransferase isoenzymes cytoplasmic BCAT1 and mitochondrial BCAT2, as well as the branched-chain alpha-keto acid dehydrogenase (BCKDH) complex [13]. The BCAT1 and BCAT2 enzymes facilitate conversion of the α-amino group of BCAAs to α-ketoglutaric acid, resulting in the production of glutamic acid (Glu) and corresponding branched-chain keto acids. It is then metabolized by the BCKDH complex to produce the tricarboxylic acid cycle intermediates acetyl-CoA and/or succinyl-CoA for energy production and macromolecular biogenesis [14]. Most cancers predominantly depend on BCAT1 (such as melanoma, breast cancer, etc.) [15–17]. Additionally, the proteins BCKDHA, BCKDHB, and others play crucial roles in the BCAA metabolic pathway [18, 19].

Recent studies have elucidated the role of BCAAs and BCAT2 in the pathogenesis of certain cancers, including pancreatic ductal adenocarcinoma (PDAC) [7, 20, 21]. Knockdown (KD) of BCAT2 has been shown to significantly impede tumor progression, while a diet low in BCAAs was reported to inhibit the development of PDAC in murine models [20]. In response to BCAA deprivation, acetylation and subsequent degradation of BCAT2 enhances cell proliferation and promotes pancreatic tumor growth [7]. Conversely, a diet high in BCAAs facilitates the progression of PDAC by stabilizing BCAT2 [21]. Although these findings provide valuable insights into the function of BCAT2 in PDAC, its role in PCa remains poorly understood.

In this study, the expression levels of BCAAs and BCAT2 in PCa samples and cell lines were evaluated, and the correlation between BCAT2 expression and clinicopathological parameters, as well as survival outcomes, of PCa patients was further investigated. Subsequently, the impact of BCAT2 KD and overexpression on PCa cell proliferation, autophagy, apoptosis, and ferroptosis was assessed, and the associated molecular mechanisms were elucidated. The findings of this study provide evidence of the biological role of BCAT2 and interaction with PCBP1, which modulates the PI3K/AKT signaling pathway. BCAT2 presents a potential therapeutic target or biomarker for PCa.

## Results

### BCAT2 expression is upregulated in PCa tissue and cells

**Fig. 1:**
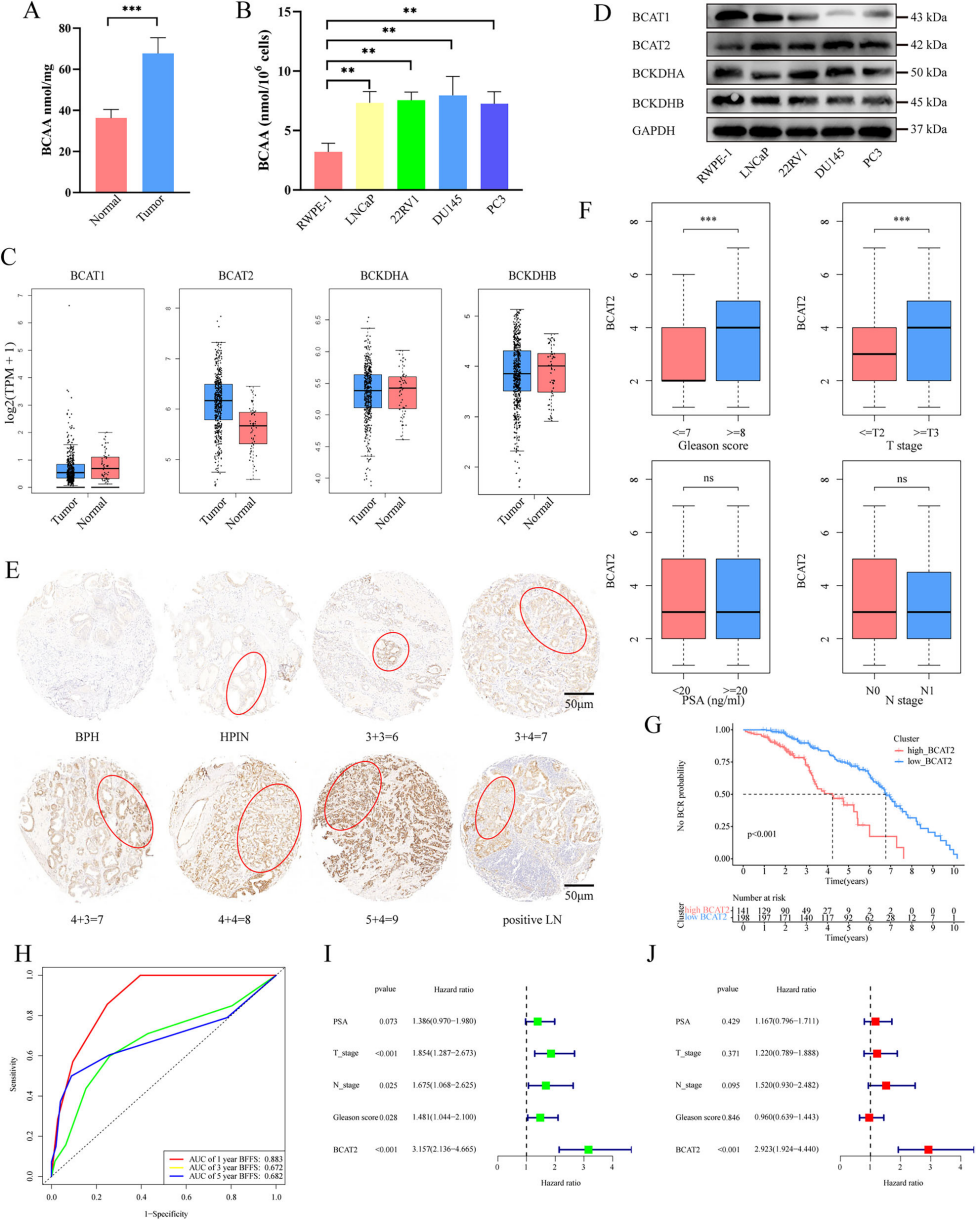
BCAT2 expression is elevated in PCa and associated with poor prognosis. **A** Analysis of BCAA levels in 10 paired tumor and normal prostate tissues (*p* < 0.001). **B** Analysis of BCAA levels in PCa cell lines (*p* < 0.01). **C** mRNA levels of BCAT1, BCAT2, BCKDHA and BCKDHB in PCa and normal tissues by GEPIA database. **D** Western blot analysis of the protein levels in PCa cell lines. **E** Immunohistochemical staining of BCAT2 in BPH and different stages of PCa. **F** IRS of BCAT2 expression in different Gleason score, T stage, N stage and PSA. **G** The KM analysis of BFFS for PCa patient. **H** Analysis of ROC curves of PCa patients with BCR at 1, 3 and 5 years. **I** Univariate and **J** multivariate Cox regression analysis of the correlation of BFFS with different clinicopathological features and BCAT2 expression in clinical cohort.

The BCAA degradation pathway plays a significant role in cancer, although relatively few studies have investigated its potential implications in PCa. Analysis of BCAA levels in 10 pairs of PCa and adjacent non-cancerous tissue samples indicated substantially higher levels in tumor tissues than paracancerous tissues (Fig. 1A). Moreover, BCAAs in PCa cell lines were significantly higher than normal prostate RWPE-1 epithelial cells (Fig. 1B).

The key enzymes involved in the BCAA degradation pathway include BCAT1, BCAT2, BCKDHA, and BCKDHB. The expression levels of these enzymes in tumor tissues are shown in Fig. S1A–D. Analysis of the Cancer Genome Atlas database revealed that only BCAT2 was significantly upregulated in PCa (Figs. 1C and S1E–H). This finding was further validated in PCa cell lines (Fig. 1D). To evaluate the clinical significance of BCAT2, immunohistochemical analysis was conducted using PCa tissue microarray chips. The results indicated that BCAT2 expression was lowest in benign prostatic hyperplasia and high-grade prostatic intraepithelial neoplasia, and progressively increased with the Gleason score. Also, BCAT2 was notably expressed in positive lymph nodes (Fig. 1E).

### BCAT2 expression is correlated with PCa progression and poor prognosis

Clinical correlation analysis showed that BCAT2 expression was positively correlated with the Gleason score and T stage (Fig. 1F). Kaplan–Meier curve analysis of 339 PCa patients revealed that high BCAT2 expression was linked to an increased risk of biochemical recurrence as compared to low BCAT2 expression (*p* < 0.001) (Fig. 1G). The area under the receiver operating characteristic curve to predict biochemical failure-free survival (BFFS) at 1, 3, and 5 years was 0.883, 0.672, and 0.682, respectively (Fig. 1H). Meanwhile, BCAT2 was a good prognostic indicator at different disease stages (Fig. S2). Univariate and multivariate Cox regression analyses were also utilized to evaluate the usefulness of BCAT2 to predict the prognosis of PCa patients. The results of univariate Cox regression analysis showed that BCAT2 expression, Gleason score, T stage, and N stage were significantly associated with BFFS (Fig. 1I), while multivariate Cox regression analysis revealed that high BCAT2 expression and lymph node involvement were independent prognostic risk factors for BFFS of PCa patients (Fig. 1J and Table S3).

### BCAT2 promotes PCa cell proliferation, migration, and invasion

**Fig. 2:**
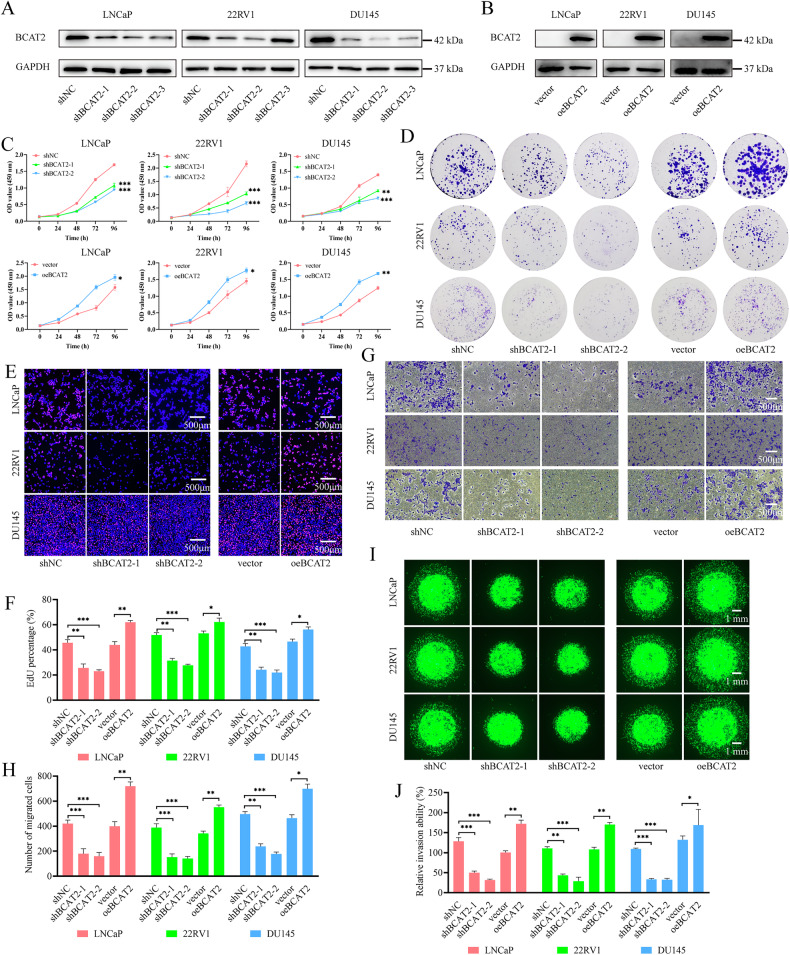
BCAT2 promotes proliferation and migration of PCa cells. **A** Expression level of BCAT2 was significantly down-regulated in BCAT2 KD PCa cells. **B** Expression level of BCAT2 was significantly up-regulated in oeBCAT2 cells. **C** CCK-8 analysis of the impact of BCAT2 knockdown or overexpression on PCa cell growth. **D** Colony formation assay investigating the effects of BCAT2 on cell proliferative. **E**, **F** EdU assay for the effect of BCAT2 on PCa cell proliferative. **G**, **H** Transwell assay for the effect of BCAT2 on PCa cell migration. **I**, **J** 3D Matrigel Drop invasion assay for the effect of BCAT2 on PCa cell invasion.

In LNCaP, 22Rv1, and DU145 cells, KD of BCAT2 was performed using shRNA lentiviruses, while an overexpression lentivirus system was employed to upregulate BCAT2 expression. Among the 3 shRNAs, shBCAT2-1 and shBCAT2-2 achieved the highest KD efficiency and, thus, were selected for further investigations (Fig. 2A, B). The results of the Cell Counting Kit-8 (CCK-8) assay, cell colony formation assay, and EdU (5-ethynyl-2’-deoxyuridine) assay demonstrated that the KD of BCAT2 significantly inhibited proliferation of PCa cells (Figs. 2C–F and S3A). Conversely, overexpression of BCAT2 markedly enhanced proliferation of PCa cells (Figs. 2C–F and S3A). Cell migration ability was assessed with both the scratch assay and Transwell assay, while invasion capability was evaluated with the three-dimensional Matrigel drop invasion assay. The results indicated that overexpression of BCAT2 enhanced both the migration and proliferation of PCa cells, whereas KD of BCAT2 had opposite effects (Figs. 2G–J and S3B, C).

### BCAT2 inhibits autophagy in PCa cells

Gene set enrichment analysis based on the RNA sequencing results of DU145-shNC and DU145-shBCAT2-2 demonstrated that BCAT2 KD activated the autophagy, apoptosis, and ferroptosis pathways (Figs. S4A, S5A, S6A). Previous studies have indicated that autophagy can promote both apoptosis and ferroptosis [22, 23]. Therefore, we hypothesized that BCAT2 facilitates apoptosis and ferroptosis of PCa cells by activating autophagy.

**Fig. 3:**
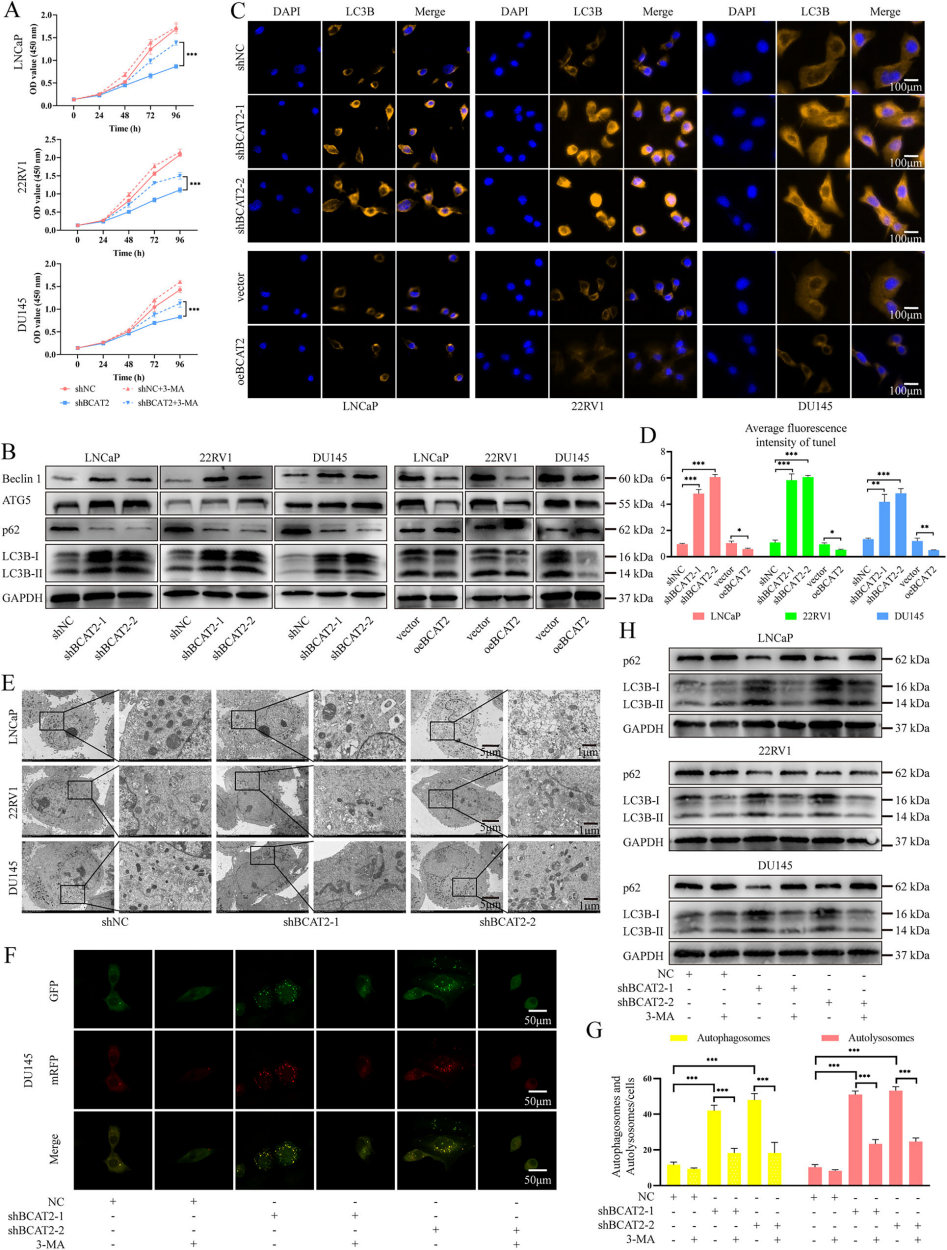
BCAT2 inhibits autophagy in PCa cells. **A** CCK-8 assay for shNC or shBCAT2-2 cells, treated with or without 5 mM 3-MA (MedChemExpress, US). **B** WB analysis of autophagy related protein levels in LNCaP, 22RV1 and DU145 cells. **C**, **D** IF staining analysis of LC3B. **E** Autophagy was evaluated with TEM in PCa shNC or shBCAT2-2 cells. **F**, **G** DU145 shNC or shBCAT2-2 cells transfected with mCherry-GFP-LC3B plasmid were treated with or without 5 mM 3-MA for 24 h. The average number of red dots (autolysosomes) and yellow dots (autophagosomes) was quantified. **H** WB analysis of indicated protein levels in shNC or shBCAT2-2 cells, treated with or without 5 mM 3-MA for 24 h.

The results of the CCK-8 assay demonstrated that the reduced proliferation of PCa cells, resulting from BCAT2 KD, was significantly restored upon the addition of the autophagy inhibitor 3-methyladenine (3-MA) (Fig. 3A). Western blot (WB) analysis of autophagy-related proteins indicated that BCAT2 KD led to increased levels of LC3B-II, ATG5, and Beclin1, and decreased levels of SQSTM1/p62 in LNCaP, 22RV1, and DU145 cells. Conversely, overexpression of BCAT2 had opposite effects (Fig. 3B). Similar results were observed by immunofluorescence staining of LC3B. The fluorescence intensity of LC3B significantly increased following BCAT2 KD and decreased after BCAT2 overexpression (Fig. 3C, D). Transmission electron microscopy indicated that BCAT2 KD elevated levels of autophagy of PCa cells as compared to negative control cells (Fig. 3E).

To further investigate the impact of BCAT2 on the autophagy process, cells were transfected with the mCherry-green fluorescent protein-LC3B dual fluorescence plasmid. The results demonstrated an increase in the quantities of both autolysosomes (yellow dots) and autophagosomes (red dots) in BCAT2-KD PCa cells as compared to control cells (Figs. 3F, G and S4B–E). This phenomenon was reversed by administration of 3-MA. Furthermore, at the protein level, 3-MA reversed the elevation of LC3B and reduction of p62 induced by BCAT2 (Fig. 3H). These findings demonstrate that BCAT2 exerts an inhibitory effect on the autophagy of PCa cells.

### BCAT2-mediated autophagy regulates caspase-dependent apoptosis of PCa cells

**Fig. 4:**
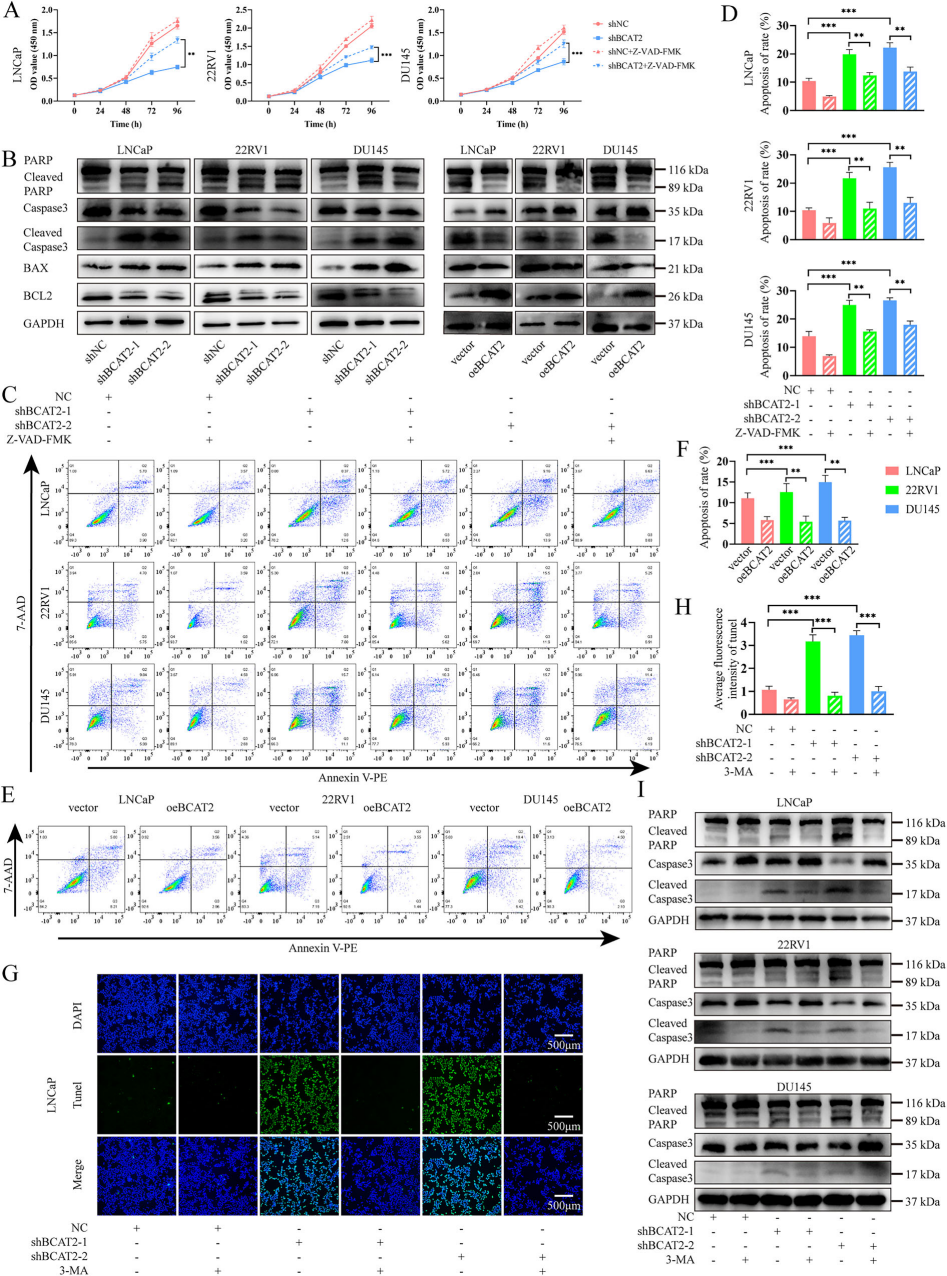
BCAT2 inhibits autophagy-related apoptosis in PCa cells. **A** CCK-8 assay for shNC or shBCAT2-2 cells, treated with or without 20 μM Z-VAD-FMK (MedChemExpress, US). **B** WB analysis of apoptosis related protein levels in PCa cells. **C**, **D** Apoptotic rate of PCa shNC or shBCAT2 cells, treated with or without 20 μM Z-VAD-FMK for 24 h, was measured with Annexin V-PE/7-AAD staining. **E**, **F** Apoptotic rate of PCa cells after BCAT2 overexpression. **G**, **H** PCa shNC or shBCAT2 cells, treated with 5 mM 3-MA for 24 h, stained with TUNEL. **I** WB analysis of apoptosis related protein levels in shNC or shBCAT2-2 cells, treated with or without 5 mM 3-MA for 24 h.

The CCK-8 assay was conducted to investigate the potential role of BCAT2 in the proliferation of PCa cells via apoptosis signaling pathways. The results indicated that the introduction of the apoptosis inhibitor Z-VAD-FMK could counteract the reduction in cell proliferation associated with BCAT2 KD (Fig. 4A). Subsequently, the expression levels of apoptosis-related proteins were assessed by WB analysis. The findings revealed that BCAT2 KD significantly decreased BCL2 expression, increased BAX levels, and triggered activation of PARP and Caspase-3. Overexpression of BCAT2 produced opposite results (Fig. 4B). Flow cytometry demonstrated a marked increase in the apoptosis rate following BCAT2 KD. However, Z-VAD-FMK significantly mitigated apoptosis induced by BCAT2 KD. Furthermore, overexpression of BCAT2 notably decreased the apoptosis rate (Fig. 4C–F).

To examine the relationship between BCAT2-mediated apoptosis and autophagy in PCa cells, BCAT2-KD cells were treated with the autophagy inhibitor 3-MA. KD of BCAT2 significantly increased the level of TUNEL staining of PCa cells. However, treatment with 3-MA mitigated BCAT2-mediated apoptosis (Figs. 4G, H and S5B–E). This finding suggests that BCAT2 KD activates autophagy of PCa cells, which was partially implicated in BCAT2-mediated apoptosis. WB analysis revealed that the levels of apoptosis-related proteins, including cleaved PARP and cleaved Caspase-3, were downregulated following 3-MA treatment (Fig. 4I). These results indicate that inhibition of autophagy reduced apoptosis of PCa cells induced by BCAT2 KD.

### BCAT2-mediated autophagy regulates ferroptosis of PCa cells

**Fig. 5:**
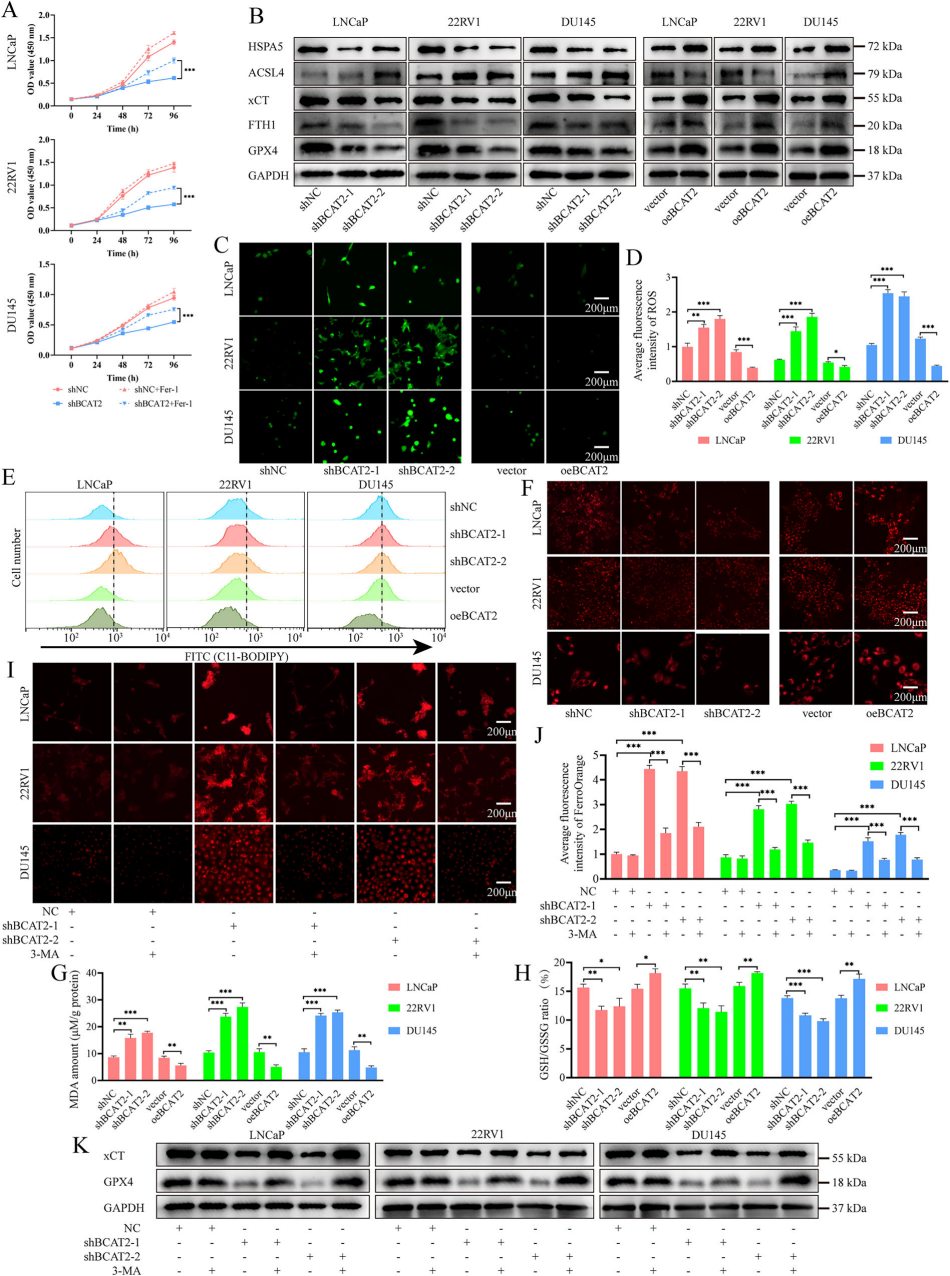
BCAT2 inhibits autophagy-related ferroptosis in PCa cells. **A** CCK-8 assay for shNC or shBCAT2-2 cells, treated with or without 2 μM Fer-1 (MedChemExpress, US). **B** WB analysis of ferroptosis related protein levels in PCa cells. **C**, **D** ROS staining of PCa cells. **E** The lipid ROS level (C11-BODIPY^®^ 581/591) of PCa cells was analyzed by a flow cytometer. **F** Mitochondrial membrane potential (TMRE staining) of PCa cells. **G**, **H** The levels of MDA and GSH/GSSG in PCa cells pretreated. **I**, **J** The levels of intracellular iron measured in PCa cells pretreated with or without 5 mM 3-MA for 24 h. **K** WB analysis of ferroptosis related protein levels in shNC or shBCAT2-2 cells, treated with or without 5 mM 3-MA for 24 h.

The CCK-8 assay demonstrated that cell death and inhibition of proliferation resulting from BCAT2 KD could be reversed by the ferroptosis inhibitor Fer-1, suggesting an increase in ferroptosis following BCAT2 KD (Fig. 5A). Subsequently, WB analysis was employed to assess alterations to the levels of ferroptosis-related proteins. The results indicated that following BCAT2 KD, the expression levels of GPX4, FTH1, xCT, and HSPA5 were significantly reduced, whereas ACSL4 expression was elevated. Corresponding outcomes were observed by overexpression of BCAT2 (Fig. 5B). Given the significant role of ROS in ferroptosis, the levels of ROS and DHE were investigated. The findings revealed that intracellular levels of ROS and DHE were markedly elevated following KD of BCAT2, but decreased by BCAT2 overexpression (Figs. 5C, D and S6B–E). Furthermore, we assessed the levels of lipid ROS and MDA, and observed that BCAT2 KD led to an increase in lipid peroxidation (Figs. 5E, G and S6F). In addition, evaluation of the impact of BCAT2 on mitochondrial membrane potential and the GSH/GSSG ratio demonstrated that the decreases in TMRE levels (Figs. 5F and S6G) and the GSH/GSSG ratio (Fig. 5H) were diminished following BCAT2 KD, but significantly elevated by BCAT2 overexpression.

To further elucidate the relationship between autophagy and ferroptosis, the cells were treated with 3-MA. The results indicated a significant increase in iron ions following BCAT2 KD, while treatment with 3-MA effectively reversed ferroptosis associated with BCAT2 KD (Fig. 5I, J). Additionally, the WB results demonstrated that 3-MA treatment counteracted the reduction in GPX and xCT levels caused by BCAT2 KD (Fig. 5K). These findings suggest that KD of BCAT2 promoted ferroptosis of PCa cells by enhancing autophagy.

### Binding of BCAT2 affects the stability of PCBP1

**Fig. 6:**
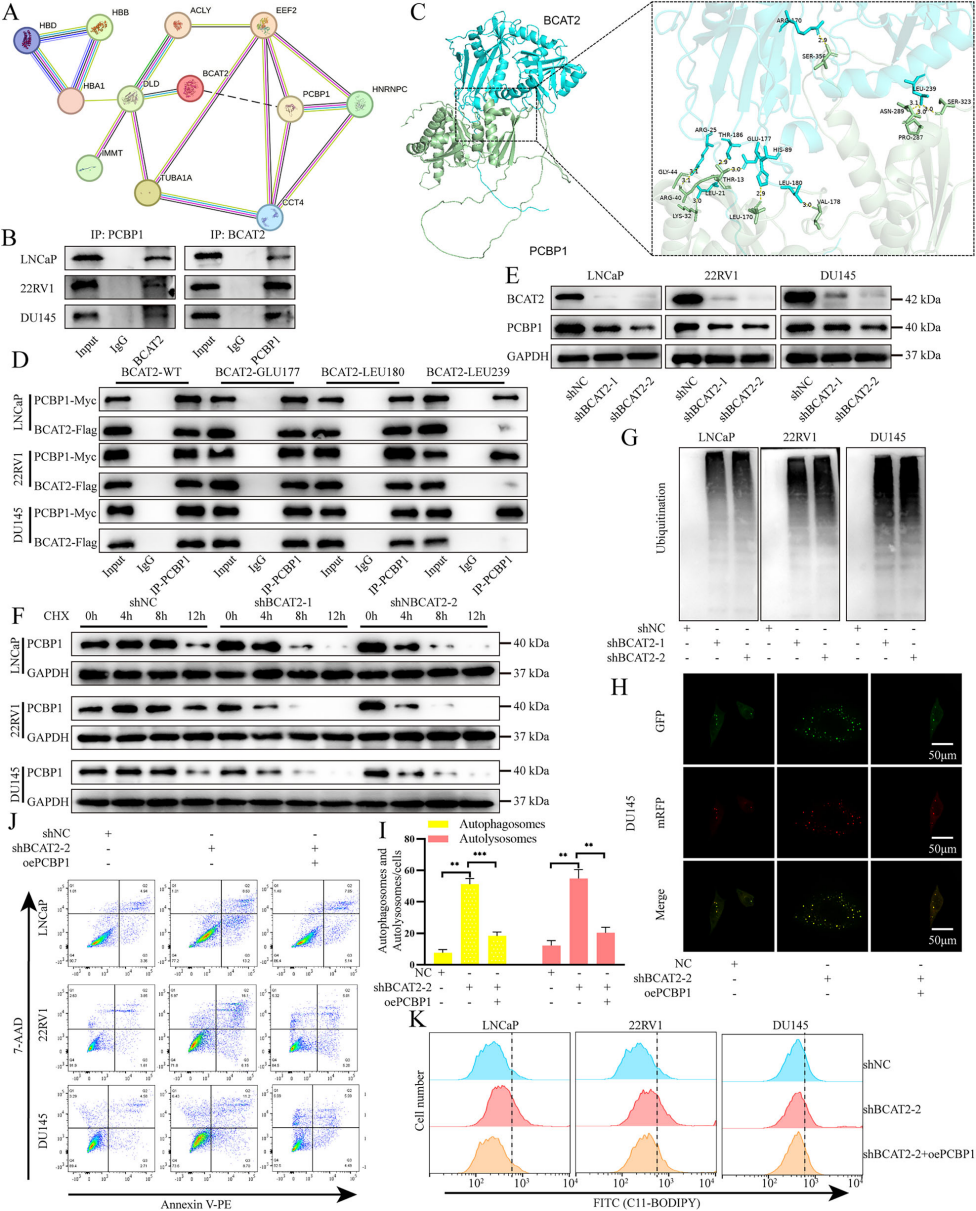
BCAT2 and PCBP1 interact to regulate the biological function of PCa. **A** PPI analysis of BCAT2-associated core proteins identified by Co-IP. **B** WB showing the association of BCAT2 with PCBP1 after Co-IP. **C** Predicted mode of binding of BCAT2 to PCBP1 based upon molecular modeling. **D** Cells were transfected with different mutations plasmids to investigate the contribution of 3 sites to the direct binding of BCAT2 and PCBP1, the BCAT2-WT was set as control. **E** WB showed that PCBP1 protein level was inhibited after BCAT2 knockdown. **F** After knocking down BCAT2, the PCBP1 protein level was detected. The cells were treated with 100 μg/ml cycloheximide (MedChemExpress, US) for specified time. **G** WB verified the effect of BCAT2 knockdown on ubiquitination level. **H**, **I** DU145 cells transfected with mCherry-GFP-LC3B plasmid, also transfected with shBCAT2 and/or oePCBP1. **J** Apoptotic rate of PCa cells, transfected with shBCAT2 and/or oePCBP1. **K** The lipid ROS level of PCa cells, transfected with shBCAT2 and/or oePCBP1.

To further investigate the molecular mechanisms of BCAT2 in PCa cells, the Co-IP assay was conducted to identify proteins associated with BCAT2. Differential bands specific to BCAT2 were observable as compared to IgG controls, and subsequently analyzed by mass spectrometry (Fig. S7A). The proteins identified as top-ranked specific interactors through mass spectrometry were subjected to PPI analysis (Fig. 6A). Notably, previous studies have indicated that PCBP1 may influence apoptosis, autophagy, and ferroptosis [24–28]. Therefore, we hypothesized that the functional effects PCBP1 are exerted by interactions with BCAT2. The interaction between BCAT2 and PCBP1 was further corroborated by WB analysis (Fig. 6B). To investigate the binding sites in greater detail, molecular docking experiments were conducted, which identified Glu177, Leu180, and Leu239 of BCAT2 as critical binding sites for PCBP1 (Fig. 6C). Thus, point mutation plasmids targeting these three sites were subsequently constructed. Co-IP analysis demonstrated that the interaction between BCAT2 and PCBP1 was significantly diminished upon mutation of Leu239 (Fig. 6D). Next, the potential impact of BCAT2 on PCBP1 protein expression was examined. The findings revealed a significant reduction in PCBP1 expression following KD of BCAT2 (Fig. 6E). To further investigate whether BCAT2 facilitates accumulation of PCBP1 by enhancing post-transcriptional stability, LNCaP, 22RV1, and DU145 cells were treated with 100 µg/ml of cycloheximide for 0, 4, 8 and 12 h, respectively. Subsequently, PCBP1 protein levels were assessed. The results indicated that the half-life of PCBP1 was significantly greater in the control cells than the BCAT2 low-expression group (Fig. 6F). Furthermore, the decrease in PCBP1 protein levels was markedly accelerated following reduced BCAT2 expression. These findings suggest that BCAT2 plays a crucial role in significantly prolonging the half-life of PCBP1 protein.

### PCBP1 overexpression can negate the impact of BCAT2 KD

Given that the functional role of BCAT2 is dependent on interactions with PCBP1, PCBP1 was overexpressed following KD of BCAT2 to determine if the function of BCAT2 could be reinstated. The cell colony formation and transwell assays demonstrated that overexpression of PCBP1 effectively counteracted the inhibitory effects of BCAT2 KD on both cell proliferation and migration (Fig. S7B–E). Furthermore, overexpression of PCBP1 mitigated autophagy, apoptosis, and ferroptosis of PCa cells as a consequence of BCAT2 KD (Figs. 6G–J and S7F–H).

### The interaction between BCAT2 and PCBP1 inhibits the PI3K/AKT pathway

**Fig. 7:**
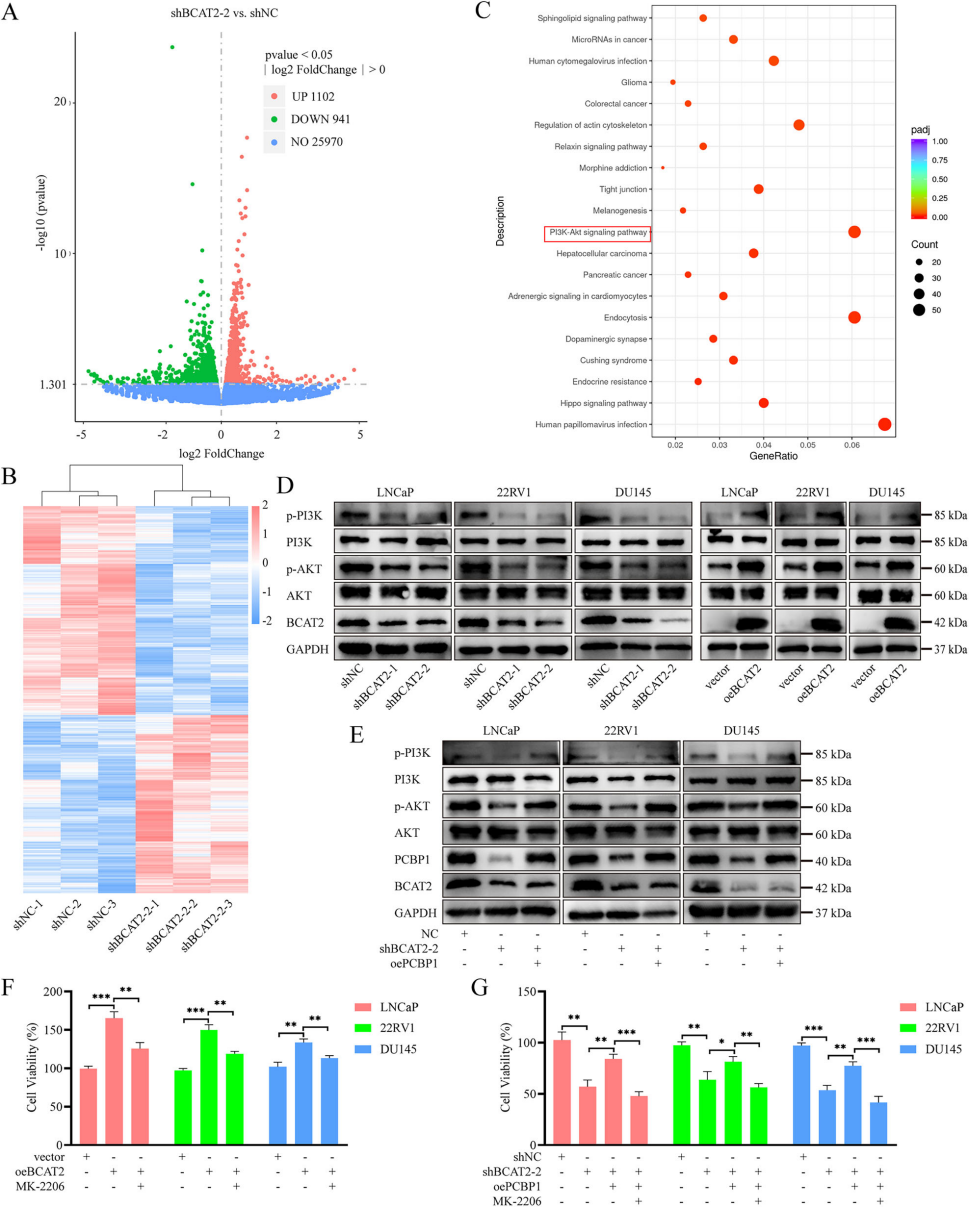
BCAT2 regulates PI3K/AKT signaling in PCa cells by binding with PCBP1. **A**, **B** Volcano and heat map illustrating differentially expressed genes in shBCAT2-2 groups and shNC groups by RNA sequencing. **C** The top 20 KEGG enriched pathways in DU145 cells with BCAT2 knockdown. **D** WB analysis of PI3K/AKT signaling pathway related protein levels in BCAT2 knockdown or overexpression PCa cells. **E** WB analysis of PI3K/AKT signaling pathway related protein levels in PCa cells transfected with shBCAT2 and/or oePCBP1. **F** Cell activity was detected in cells overexpressed BCAT2, treated with or without PI3K/AKT pathway inhibitor MK-2206. **G** Cell activity was detected in cells transfected with shBCAT2 and/or oePCBP1, treated with or without PI3K/AKT pathway inhibitor MK-2206.

To further investigate the potential signaling pathway of BCAT2 in PCa, comparative analysis of differentially expressed genes and pathways between the shNC and shBCAT2-2 groups was conducted with RNA sequencing and Kyoto Encyclopedia of Genes and Genomes (KEGG) enrichment analysis. The results indicated that the PI3K/AKT signaling pathway was significantly downregulated in the shBCAT2-2 group (Fig. 7A–C). WB analysis demonstrated that KD of BCAT2 decreased phosphorylation of AKT and PI3K in LNCaP, 22RV1, and DU145 cells, without affecting the total protein levels. Conversely, overexpression of BCAT2 increased phosphorylation of both AKT and PI3K (Fig. 7D). Furthermore, WB analysis was conducted to determine whether the PI3K/AKT pathway is influenced by interactions with PCBP1. The results revealed that overexpression of PCBP1 in PCa cells counteracted the reduction in AKT and PI3K phosphorylation induced by BCAT2 KD, while total protein levels remained relatively unchanged (Fig. 7E). Additionally, the AKT inhibitor MK-2206 reversed proliferation of PCa cells stimulated by BCAT2 overexpression (Fig. 7E). Simultaneously, PCBP1 mitigated the proliferation inhibition caused by BCAT2 KD, a phenomenon that was also reversed by MK-2206 (Fig. 7G). These findings suggest that the interaction between BCAT2 and PCBP1 modulates the PI3K/AKT signaling pathway, which plays a significant role in the progression of PCa.

### BCAT2 inhibits the progression of PCa in vivo

**Fig. 8:**
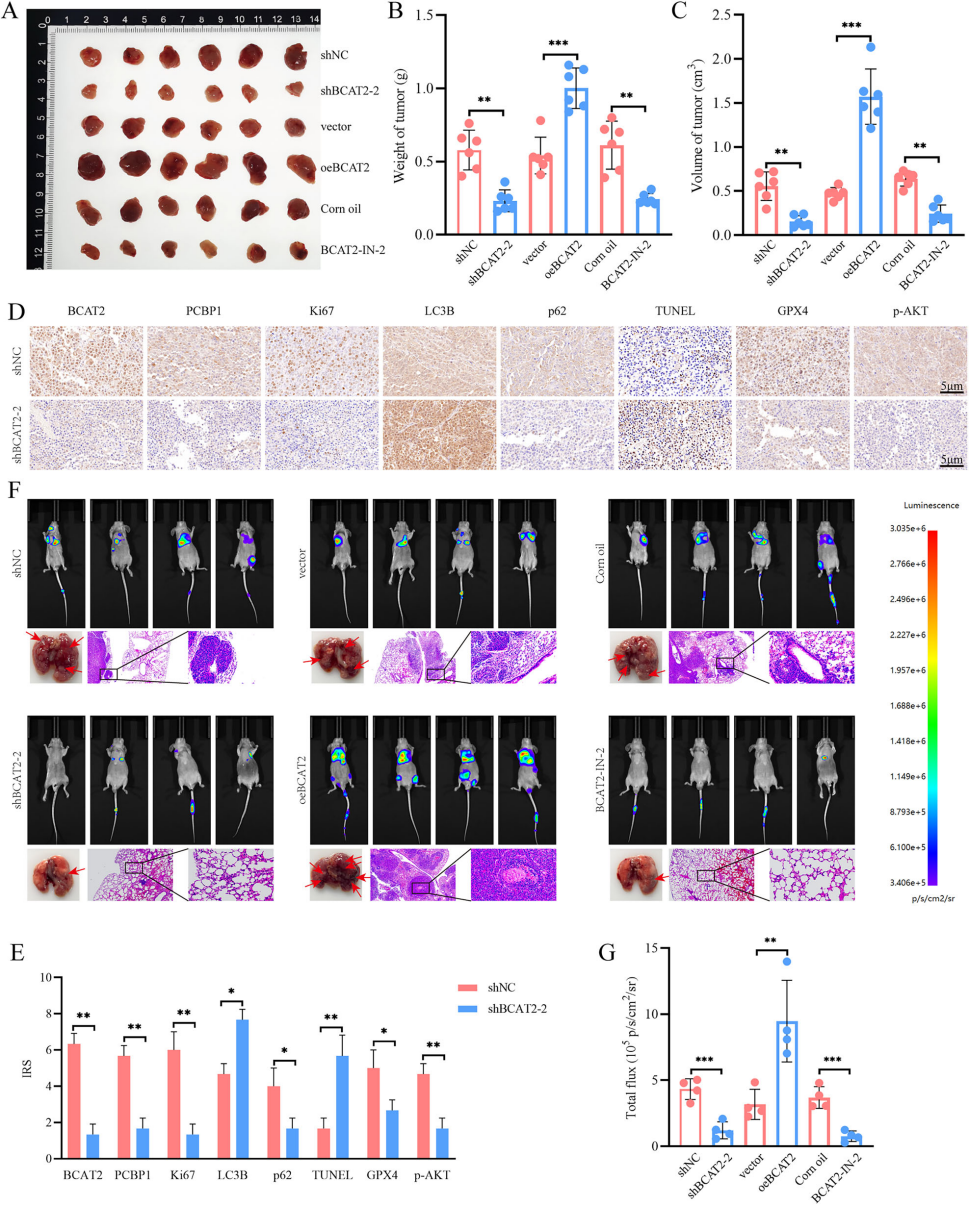
The role of BCAT2 in vivo. **A** Different groups of DU145 cells were injected subcutaneously into nude mice. **B** Tumor weight and **C** tumor volume were statistically analyzed. **D**, **E** Immunohistochemical staining was performed to detect markers of cellular proliferation, autophagy, apoptosis, ferroptosis and p-AKT activity in the tumor of mice. **F**, **G** In vivo imaging of different groups of luciferase labeled PCa lung metastasis models, as well as representative lung tissue and hematoxylin-eosin staining. The fluorescence intensity of each model was obtained under the same exposure.

To further elucidate the role of BCAT2 in vivo, nude mice were subcutaneously injected with transfected and wild-type DU145 cells. The wild-type groups were administered either pure corn oil or BCAT2-IN-2. The results indicated a significant reduction in tumor volume and quality following BCAT2 KD, whereas BCAT2 overexpression resulted in a marked increase in these parameters (Figs. 8A–C and S8A). Additionally, treatment with BCAT2-IN-2 substantially decreased both tumor volume and mass (Figs. 8A–C and S8A). Immunohistochemical staining revealed a significant reduction in PCBP1 expression after BCAT2 KD. The observed decrease in Ki67 protein levels suggests that BCAT2 plays a role in promoting cell proliferation. Furthermore, an increase in LC3B protein levels and a decrease in p62 protein levels indicate that BCAT2 may inhibit autophagy. TUNEL staining showed an increase, while GPX levels decreased, suggesting that BCAT2 inhibits both apoptosis and ferroptosis. Notably, p-AKT protein levels were significantly reduced, indicating that BCAT2 may promote AKT phosphorylation (Fig. 8D, E). These findings were reversed in the BCAT2 overexpression group (Fig. 8D, E), while consistency was observed in the BCAT2-IN-2 group (Fig. S8B–E), in agreement with the results of previous in vitro experiments.

To further evaluate the impact of BCAT2 on PCa metastasis, mice were injected via the tail vein with transfected and wild-type DU145 cells. Also the wild-type groups were administered either pure corn oil or BCAT2-IN-2. Bioluminescence imaging conducted at 4 weeks post-inoculation revealed significantly diminished transfer signals in the BCAT2 KD and BCAT- IN-2 groups, whereas BCAT2 overexpression resulted in increased transfer signals (Fig. 8F, G). Following sacrifice of the mice, lung tissues were harvested for hematoxylin and eosin staining. The results demonstrated that elevated expression of BCAT2 was associated with an increased risk of PCa metastasis to the lungs.

## Discussion

In this study, a significant increase in BCAT2 expression in PCa was initially observed, which was strongly correlated with patient outcomes. Upregulation of BCAT2 appears to play a carcinogenic role in PCa by promoting tumor cell proliferation, invasion, and migration. Furthermore, we demonstrated that BCAT2 regulates the PI3K/AKT signaling pathway by interacting with PCBP1, thereby influencing autophagy-related apoptosis and ferroptosis of PCa cells. BCAT2 KD and overexpression cell lines were constructed by lentiviral transfection, achieving substantial KD efficiency. To address the limitations associated with off-target effects of shRNA, two distinct shRNA sequences and three cell lines were selected for repeated validation, while overexpressed BCAT2 was utilized in rescue experiments. To confirm the role of BCAT2 in vivo and enhance the clinical relevance, BCAT2-IN-2, a specific inhibitor of BCAT2, was employed to validate the findings with an in vivo model. The results showed that BCAT2 interacts with PCBP1 at a specific site and co-regulates the PI3K/AKT pathway, playing a critical role in the initiation and progression of PCa. Hence, targeting BCAT2 presents a promising therapeutic strategy to inhibit progression of PCa.

Amino acid metabolism is integral to the development and progression of cancer [29]. BCAAs (Leu, Ile, and Val) are essential for protein synthesis and various metabolic pathways. In cancerous tissues, uptake and metabolism of BCAAs are typically elevated to support rapid cellular growth and proliferation [6]. An increase in BCAT1/2 expression facilitates a reversible reaction between BCAAs and α-ketoglutaric acid, yielding alpha-ketoic acids and Glu, respectively [14]. In numerous tumors, including bladder cancer, melanoma, and glioma, enhanced expression of BCAT1 is associated with tumor progression and patient prognosis. Furthermore, targeted inhibition of BCAA metabolism and BCAT1 has been shown to impede tumor progression [30–35]. Conversely, in a limited number of tumors, such as pancreatic and lung cancers, BCAT2 appears to play a more significant role [7, 20, 36].

Research investigating the role of BCAT2 in cancer has unveiled a more intricate mechanism. Mutations to the KRAS gene, particularly in pancreatic and lung tissues, establish a distinct reliance on BCAA metabolism. PDAC cells exhibit diminished uptake of BCAAs, whereas non-small cell lung cancer cells absorb and utilize free BCAAs as a nitrogen source [36]. Furthermore, several studies suggest that BCAT2 may facilitate oncogenesis of PDAC and KD of BCAT2 significantly impaired proliferation of PDAC cells [20]. Additionally, BCAT2-mediated BCAA catabolism is known to enhance mitochondrial respiration and is crucial for progression of PDAC associated with KRAS mutations [20, 37]. Notably, increasing dietary BCAA intake has been linked to progression of PDAC. Moreover, both BCAAs and BCAT2 have been implicated in regulation of apoptosis, autophagy, and ferroptosis [38–41].

PCBP1, an RNA-binding protein, plays a significant role in various cellular processes, including gene transcription, RNA regulation, ferroptosis, and autophagy [42]. Notably, PCBP1 has been shown to impede autophagic flux in tumor cells. Mechanistically, overexpression of PCBP1 inhibits expression of LC3B, ultimately leading to reduced autophagy [26]. Autophagy is essential for removal of damaged organelles and proteins, thereby protecting cells from the accumulation of harmful substances and mitigating cellular damage. However, excessive autophagy can trigger apoptosis, a phenomenon referred to as autophagy-related apoptosis [22]. Recent studies have indicated that ferroptosis can manifest as a form of autophagy-dependent cell death. Autophagy disrupts the redox balance and promotes ROS-dependent ferroptosis, while ROS generated during ferroptosis can further induce autophagy, creating a positive feedback loop [23]. This study is the first to report that BCAT2, in conjunction with PCBP1, regulates the PI3K/AKT signaling pathway, thereby facilitating progression of PCa.

In conclusion, the results of this study indicate that upregulated BCAT2 expression is correlated with a poor prognosis of PCa. The Leu239 site of BCAT2 plays a regulatory role in the PI3K/AKT signaling pathway through interactions with PCBP1, thereby inhibiting autophagy-related apoptosis and ferroptosis of PCa cells. Consequently, BCAT2 presents a potential diagnostic and prognostic biomarker, as well as a promising therapeutic target for PCa.

## Methods and materials

### Clinical samples

Tissue specimens were obtained from patients with primary PCa who underwent surgical resection at Shanghai East Hospital Affiliated to Tongji University. PCa tissue microarrays were obtained from Fudan University Shanghai Cancer Center, which included 339 PCa samples and 20 benign prostatic hyperplasia samples. The clinical data of these PCa patients were obtained from medical records and included follow-up data (i.e., age, Gleason score, prostate specific antigen levels, tumor stage, biochemical recurrence, etc.).

### BCCA assay

BCAA kits (ab83374; Abcam Limited, Cambridge, UK) were used to detect BCAA levels in the samples. In brief, samples and working solutions were prepared in accordance with the manufacturer’s protocols and standard curves were generated from 96-well plates using a microplate reader (SpectraMax iD5; Molecular Devices, San Jose, CA, USA). BCAA levels were determined in reference to the standard curve.

### Cell culture

PCa cells (LNCaP, 22RV1, PC3 and DU145), 293 T cells, and normal prostate RWPE-1 epithelial cells were acquired from the Chinese Academy of Sciences Committee Typical Culture Collection Cell Bank (Shanghai, China). The PCa cell lines were cultured in Roswell Park Memorial Institute 1640 medium containing 10% fetal bovine serum (FBS; Gibco; Thermo Fisher Scientific, Waltham, MA USA) and 1% penicillin-streptomycin (PS; Gibco), while HEK293T cells were cultured in Dulbecco’s modified Eagle’s medium supplemented with 10% FBS and 1% PS, and RWPE-1 cells were grown in keratinocyte-serum-free complete medium (Thermo Fisher Scientific). All cells were cultured in a cell incubator at 37 °C under an atmosphere of 5% CO_2_/95% air.

### Western blot (WB) analysis

Total cellular proteins were extracted using radio-immunoprecipitation assay buffer (Epizyme, Shanghai, China) containing protease and phosphatase inhibitors. A bicinchoninic acid protein assay kit (Epizyme) was utilized to measure the protein concentrations. The proteins were separated by sodium dodecyl sulfate-polyacrylamide gel electrophoresis and electroblotted on to nitrocellulose membranes, which were blocked with 5% skim milk for 1–2 h at room temperature and probed overnight at 4 °C with primary antibodies followed by secondary antibodies for 1 h at room temperature. Then, the protein bands were visualized with an Amersham Imager 600 (GE HealthCare Technologies, Inc., Chicago, IL, USA). The primary antibodies are listed in Table S1.

### Immunohistochemical analysis

The tissue chips were heated at 59 °C in an incubator for 60 min, soaked in different concentrations of xylene and ethanol solutions for 10 min, respectively, then soaked in distilled water for 10–15 min, rinsed, soaked with 200 ml of 3% H_2_O_2_ and 1 ml of NaN_3_ for 10 min, and rinsed. After antigen repair, the chips were incubated with an appropriate primary antibody overnight at 4 °C, followed by a second antibody for 35 min at 37 °C. Following coloration, dehydration, and sealing, the staining intensity was scored by experienced pathologists in accordance with the World Health Organization histological classification criteria for PCa [43] as 0 (negative), 1 (weak), 2 (medium), or 3 (strong). The positive cell rate was scored as 1 (0%–25%), 2 (26%–50%), 3 (51%–75%), or 4 (76%–100%). A total immunoreactive score <4 was defined as low expression and ≥4 as high expression.

### Cell transfection

The sequences of short hairpin RNA (shRNA) against BCAT2 (shBCAT2) and a negative control (shNC) were encoded within a PGMLV-hU6-MCS-CMV-Puro vector (Genomeditech, Shanghai, China) (Table S2). For gene overexpression, the PGMLV-CMV-MCS-3×Flag-PGK-Puro/Neo vector (Genomeditech, Shanghai, China) was subcloned with BCAT2 (oeBCAT2) and PCBP1 (oePCBP1), while an empty vector was used as a negative control. The interfering plasmid was packed in HEK293T cells at lentiviral construct, packaging plasmid, and envelope plasmid ratios of 1 µg:900 ng:100 ng. The viral supernatants were collected at 48–72 h after transfection. LNCaP, 22Rv1, and DU145 cells were transfected with the lentivirus vector and screened with 2 µg/ml of puromycin (Genomeditech, Shanghai, China) or 400 µg/ml of G418 Sulfate (Genomeditech) for 2 weeks. Subsequently, surviving cells were cultured for the following experiments. Transfection efficiency was validated by WB analysis.

### Cell viability assay

Cells (3 × 10^3^) were seeded into the wells of 96-well plates, with 3 replicate wells for each group, and incubated at 37 °C under an atmosphere of 5% CO_2_ for the appropriate time. Then, 10 μl of CCK-8 reagent (Epizyme) were added to each well and incubation was continued for 2 h. The absorbance of cells incubated for different times at 450 nm were measured with a microplate reader.

### Cell colony formation assay

Cells (1 × 10^3^) were evenly spread in each well of six-well plates and cultured at 37 °C under an atmosphere of 5% CO_2_ for 2 weeks until colonies were formed. Afterward, the colonies were fixed with 4% paraformaldehyde solution (Servicebio, Wuhan, Shanghai) for 30 min at room temperature and stained with 0.5% crystal violet (Beyotime, Shanghai, China) for 10 min.

### EdU assay

Cells (3 × 10^4^) were seeded into the wells of 96-well plates in advance. Then, 100 µl of medium with 50 μM EdU reagent (Ribobio, Guangzhou, China) were added to each well and the plate was incubated for 2 h. After fixation with 4% poly-methanol for 30 min, the plates were incubated with 0.5% TritonX-100 for 10 min. Subsequently, 1X Apollo® staining reaction and Hoechst 33342 reaction solution were added and incubation was continued for 30 min. After discarding the staining solution, the wells were imaged under a fluorescence inverted microscope (Nikon Corporation, Tokyo, Japan). All procedures were performed in the dark at room temperature.

### Cell migration assay

The transwell assay was performed with a 24-well transwell chamber with 8-µm well membranes (Corning Inc., Corning, NY, USA). Cells (3 × 10^4^) were cultured in serum-free medium for 24 h in the upper chamber or cultured for 48 h with medium containing 20% FBS in the lower chamber. After fixation with 4% paraformaldehyde, cells in the lower chamber were stained with 0.05% crystal violet for 10 min. The chambers were rinsed with clean water to remove the upper layer cells and dried at room temperature. Three randomly selected fields were imaged under an inverted light microscope (Nikon) and the number of cells passing through the lower layer of the microporous membrane was counted.

### Wound healing assay

Cell monolayers in 6-well plates at 80% confluence were scratched with a 200-µl pipette tip. The cells were cultured in fresh serum-free medium at 37 °C under an atmosphere of 5% CO_2_. Images of migrating cell were obtained at the same locations at 0 and 24 h using an inverted microscope.

### Three-dimensional Matrigel drop invasion assay

Cells (5 × 10^4^) were mixed with Matrigel and seeded into the wells of 24-well plates on ice. After placing the plate upside down in an incubator at 37 °C for 10 min, medium containing 20% FBS was added and incubation was continued for 1 week. Following the addition of 250 μl of Calcein AM solution (Beyotime), incubation was continued at 37 °C in the dark for 30 min to allow viable cells to emit green fluorescence. Images were acquired using a fluorescence inverted microscope.

### Annexin V-phycoerythrin (PE)/7-aminoactinomycin D (7-ADD) double staining

For apoptosis detection, cells were processed in advance in 6-well plates. Floating cells in the wells were harvested, while the remaining cells were digested, harvested with trypsin (Gibco), resuspended, and stained with binding buffer in accordance with the instructions of the Annexin V-PE/7-AAD Apoptosis Detection Kit (Vazyme, Nanjing, China). The proportion of apoptotic cells was determined by flow cytometry (BD FACSCanto II; BD Biosciences, Franklin, NJ, USA).

### Terminal deoxynucleotidyl transferase dUTP nick end labeling (TUNEL) assay

Cells were added to the wells of 96-well plates in advance and treated accordingly. Then, cells were fixed with 4% paraformaldehyde for 30 min and incubated with 0.5% Triton X-100 for 5 min at room temperature. After the addition of 50 μl of TUNEL solution (Beyotime), incubation was continued for 60 min at 37 °C in the dark. Then, 50 μl of 4′,6-diamidino-2-phenylindole solution (Beyotime) were added and incubation was continued in the dark for 5 min at room temperature. Finally, the cells were imaged under a fluorescence inverted microscope.

### Immunofluorescence

The cells were spread on cell climb sheets, then fixed, and permeabilized. Bovine serum albumin (2%; Gibco) was added to the climbing tablets and blocked for 60 min at room temperature. The cells were incubated with appropriate primary antibodies overnight at 4 °C, followed by corresponding secondary antibodies at 37 °C in the dark for 1 h. After incubation for 10 min at room temperature with 4′,6-diamidino-2-phenylindole staining solution, the slides were sealed using mounting solution with anti-fluorescence quencher and imaged under a fluorescence microscope or confocal microscope (Carl Zeiss AG, Jena, Germany).

### Transmission electron microscopy

Cells were collected, fixed overnight with precooled 2.5% glutaraldehyde at 4 °C, fixed with 1% osmium tetroxide for 2 h at 4 °C, dehydrated in a 50%–100% gradient of ethanol solution, and subsequently embedded in Epon812 epoxy resin. Ultrathin sections around 60 nm were cut with a microtome and stained with uranyl acetate and lead citrate, and finally observed under a transmission electron microscope (TEM, JEM-F200, Japan).

### Autophagic flow monitoring

Target cells were transfected with the cytomegalovirus (CMV)-mCherry-green fluorescent protein-LC3B vector (Beyotime) using Lipomaster 3000 Transfection Reagent (Vazyme). After 48 h of transfection, images were acquired and analyzed under a confocal microscope. The mean number of yellow spots (autophagosomes) and red spots (autolysosomes) in each cell fusion image was quantified with a dual fluorescence system.

### Reactive oxygen species (ROS) assay

Cells were added to the wells of 96-well plates in advance. After the culture medium was removed, the cells were incubated with an appropriate volume of 2′,7′-dichlorodihydrofluorescein diacetate (Thermo Fisher Scientific) diluted to 10 μM in serum-free medium in an incubator for 20 min. After thorough washing with serum-free cell culture medium, the cells were observed under a fluorescence inverted microscope.

### Dihydroethidium (DHE) assay

DHE (MedChemExpress, Monmouth Junction, NJ, USA) was used as a fluorescent probe to detect superoxides, which indirectly reflects ROS levels in cells. The cells were incubated with 10 μM DHE solution at 37 °C for 30 min and then directly observed under a fluorescence inverted microscope.

### Lipid ROS assay

For detection of lipid ROS levels, cells were processed in advance in the wells of 6-well plates. Before detection, the cells were incubated with 10 μM C11-BODIPY^®^ 581/591 (Abclonal, Wuhan, China) for 1 h. After removal of excess dye, the cells were digested with trypsin and resuspended in phosphate-buffered saline containing 5% FBS before detection by flow cytometry.

### Malondialdehyde (MDS)

MDA levels were measured using a MDA assay kit (Beyotime). The samples and working solutions were prepared as described in the instructions. The MDA content in the sample solution was calculated in reference to a standard curve and expressed as the protein content per unit weight (μmol/mg protein).

### Mitochondrial membrane potential assay

The tetramethylrhodamine ethyl ester (TMRE) kit (Beyotime) was used to measure the mitochondrial membrane potential of PCa cells. TMRE working solution was added to well-adherent cells and incubated at 37 °C for 30 min in a cell incubator. After removal of excess dye, the cells were observed under a fluorescence inverted microscope.

### Reduced (GSH)-to-oxidized (GSSG) glutathione ratio

The GSH and GSSG assay kit (Beyotime) was used to measure the contents of reduced and oxidized glutathione. Briefly, fresh cells or tissue samples were prepared in accordance with the manufacturer’s protocols, and standards and working solutions were prepared. The samples or standards were added to the wells of 96-well plates, and the contents of total glutathione, GSSG, and GSH were determined using a microplate reader by reference to standard curves.

### Ferric ion assay

FerroOrange (Dojindo Laboratories, Mashiki, Japan) was utilized to evaluate intracellular ferric ion levels. The prepared cells were incubated with 1 μmol/l of FerroOrange working solution in serum-free medium at 37 °C for 30 min under an atmosphere of 5% CO_2_ and then immediately observed under a fluorescence inverted microscope.

### Co-immunoprecipitation (Co-IP)

The Co-IP assay was performed using a Co-IP kit (Epizyme). In brief, the cells were lysed on ice, the supernatant was centrifuged (12,000 rpm, 30 min, 4 °C), and the protein content was collected. An appropriate amount of protein A/G agarose resin was put into a centrifuge tube, resuspended in buffer, and centrifuged at 500 rpm for 1 min. The lysate incubated with the antibody was added to the cleaned resin while mixing for 1 h at room temperature, then centrifuged at 500 rpm for 1 min. Following aspiration of the supernatant, the resin was resuspended in wash buffer, centrifuged at 500 rpm for 1 min (repeated once), and heated at 95 °C for 10 min to denature the proteins for subsequent experiments. The input was used as a positive control and IgG as a negative control.

### RNA sequencing

Total RNA was extracted from shNC and shBCAT2-2 cells with TRIzol reagent (Epizyme). DNA libraries were constructed using Oligo beads and AMPure Xpbeads. The Qubit2.0 Fluorometer was used for initial quantification, the library was diluted to 1.5 ng/μl, and the insert size of the library was subsequently checked using an Agilent 2100 bioanalyzer to ensure quality. Illumina sequencing was subsequently performed and 150-bp paired-end reads were generated. HISAT2 v2.0.5 was used to construct the index of the reference genome, as well as to align the clean sequences at both ends with the reference genome.

### Mass spectrometry

Proteins collected by Co-IP were separated and subsequently stained with Coomassie Blue Fast Staining and No-decoloring Solution (Epizyme). Differential gel bands specific for BCAT2 were identified by mass spectrometry using a Q Exactive^TM^ HF-X Mass Spectrometer (Thermo Fisher Scientific). The top 30 proteins with the highest BCAT2 group specific expression, as determined by mass spectrometry, were subjected to protein–protein interaction (PPI) analysis. Then, the isolated proteins in the network were removed and only the core network was retained.

### Protein docking assay

The HDOCK server (http://hdock.phys.hust.edu.cn/) was used to model molecular docking of BCAT2 (Uniprot ID: O15382) and PCBP1 (Uniprot ID: Q15365). The docking score, confidence score, and ligand root-mean-square deviation were used as the docking evaluation criteria to identify the 10 best docking positions. The model with the highest score was selected as the best docking model. PyMol 2.4 software (https://pymol.org/) was used to visualize the results.

### Point mutation

Site-directed mutagenesis was performed using the Mut Express II Rapid Mutagenesis Kit V2 (Vazyme) to generate Flag-BCAT2-GLU177, Flag-BCAT2-LEU180, and Flag-BCAT2-LEU239.

### Animal models

Male BALB/c nude mice (age, 4 weeks) were purchased from Beijing Vital River Laboratory Animal Technology (Beijing, China) and inoculated subcutaneously via the left armpit with the designated cells (2 × 10^6^). BCAT2-IN-2 (MedChemExpress), a specific inhibitor of BCAT2, was dissolved in corn oil. The mice were gavaged with BCAT2-IN-2 at 100 mg/kg for 48 h, while control mice were gavaged with only corn oil. After 2 weeks, the mice were sacrificed and xenograft tumors were resected for subsequent experiments.

For mouse lung metastasis models, each mouse was injected via the tail vein with luciferase-treated cells (2 × 10^6^). Upon observing significant weight loss over approximately 1 month, a real-time imaging system (AniView100, Guangzhou, China) was employed to capture and analyze differences in lung metastases among the various treatment groups.

### Bioinformatics analysis

BCAT2 mRNA expression in PCa and clinical data were obtained from the Cancer Genome Atlas database (https://cancergenome.nih.gov/) [44]. The expression of proteins related to BCAA metabolism in PCa and normal tissue samples was analyzed by Gene Expression Profiling Interactive Analysis (http://gepia.cancer pku.cn/) [45]. BCAT2 expression in pan-carcinoma tissues was obtained from the UALCAN database [46]. Kaplan–Meier curves and univariate and multivariate Cox models were utilized for survival analysis. Differentially expressed mRNAs were screened with the R package DESeq2 (version 1.20.0) according to *p* < 0.05. KEGG analysis (https://www.genome.jp/kegg/) was used to identify the biological functions of BCAT2 [47]. Gene set enrichment analysis (version 4.1.0) and the molecular signatures database (version 7.5.1) were also utilized to assess the differential pathways between different groups [48]. PPI analysis was performed using the STRING database [49].

### Statistical analysis

The data were analyzed with either R 4.1.1 or Prism 9.0 software (GraphPad Software, LLC, San Diego, CA, USA). Statistical differences were analyzed with the two-tailed Student’s *t* test or one-way analysis of variance. The association between BCAT2 expression and clinicopathological features was evaluated with the *χ*^2^ test. Quantitative analysis of the images was performed using ImageJ software (https://imagej.net/ij/). The flow cytometry data were analyzed with FlowJo V10 software (https://www.flowjo.com/). A probability (*p*) value < 0.05 was considered statistically significant. (ns: *p* > 0.05; *p < 0.05; **p < 0.01; ***p < 0.001).

## Supplementary information

Supplementary original blots

Supplementary information

## Data Availability

The data that support the findings of this study are available from the corresponding author upon reasonable request.
