# Supplementary material for: BCAT2 binding to PCBP1 regulates the PI3K/AKT signaling pathway to inhibit autophagy-related apoptosis and ferroptosis in prostate cancer
Source: Cell Death Dis. 2025 Apr 24;16(1):337. doi: 10.1038/s41419-025-07559-3 (PMC12022009; doi:10.1038/s41419-025-07559-3)

Figure 1

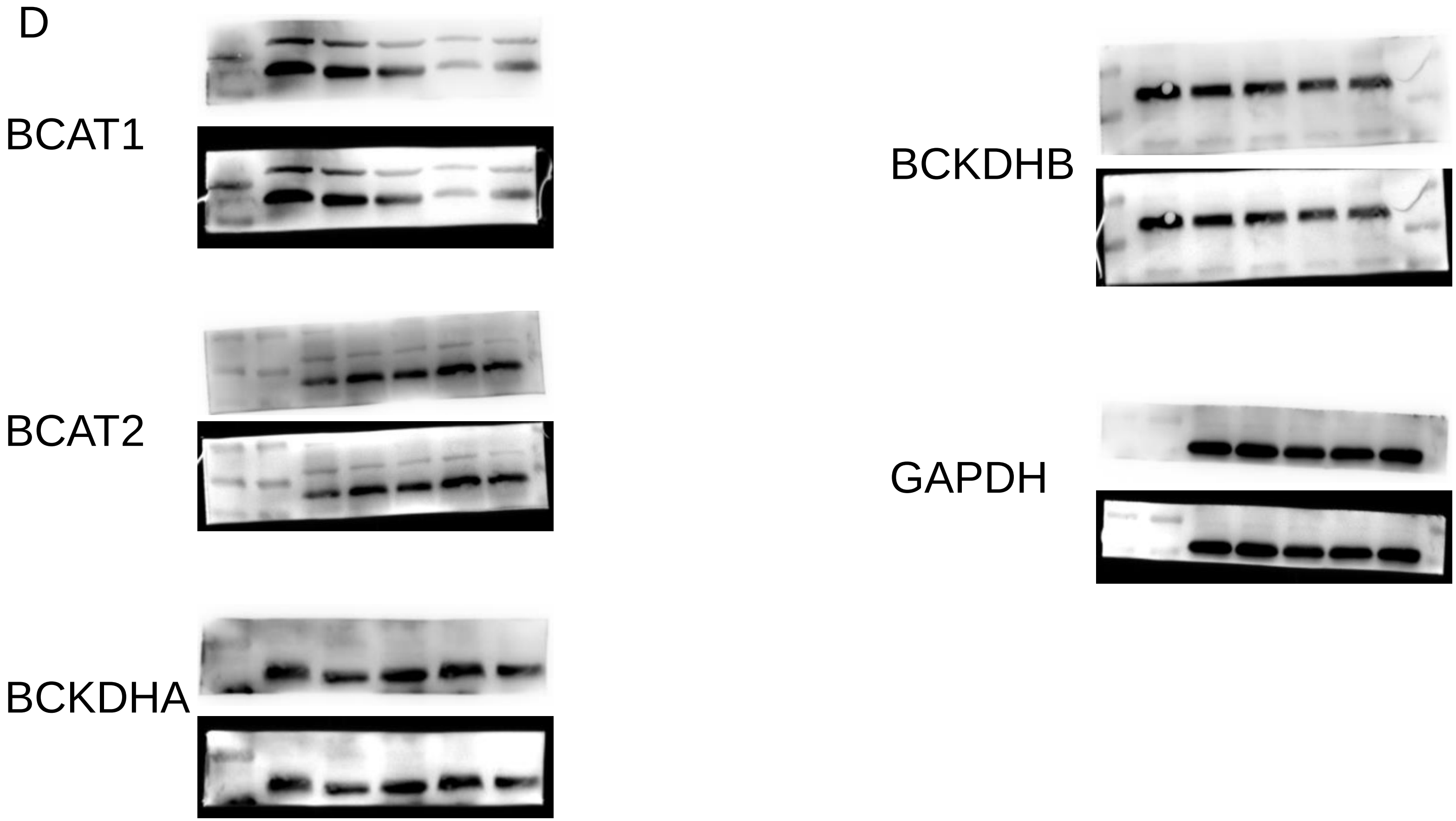

Figure 2

A

BCAT2

GAPDH

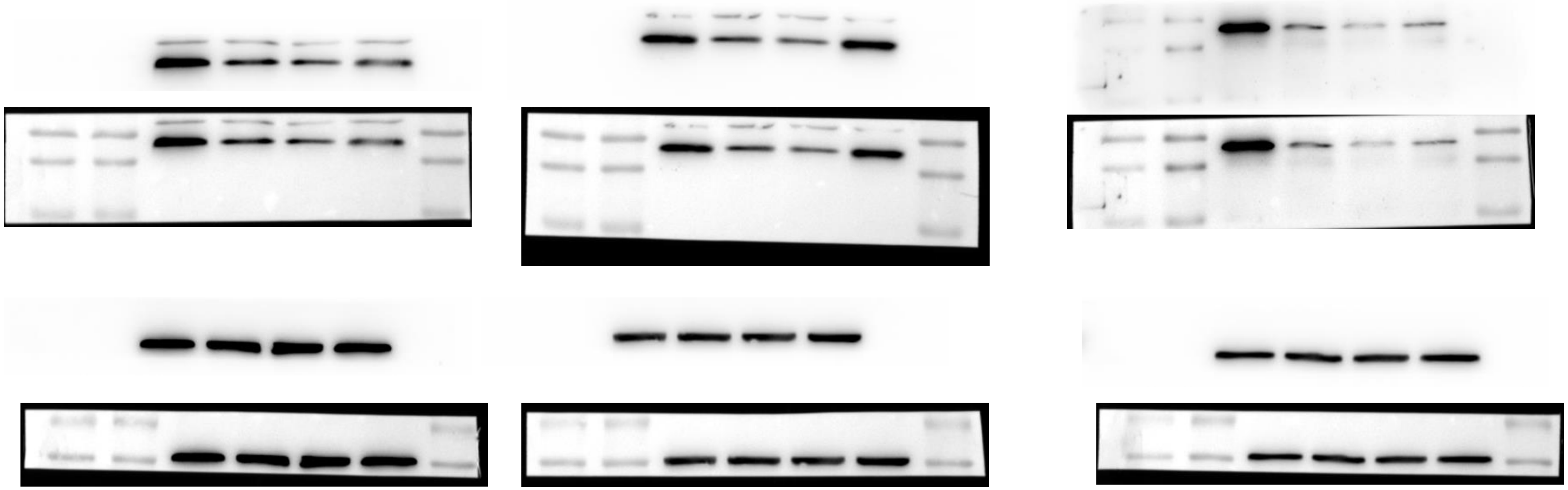

B

BCAT2

GAPDH

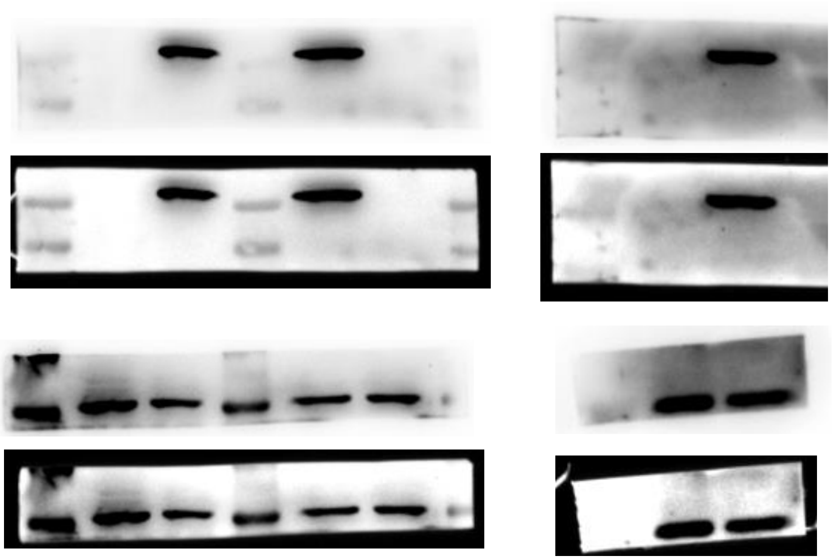

Figure 3

B

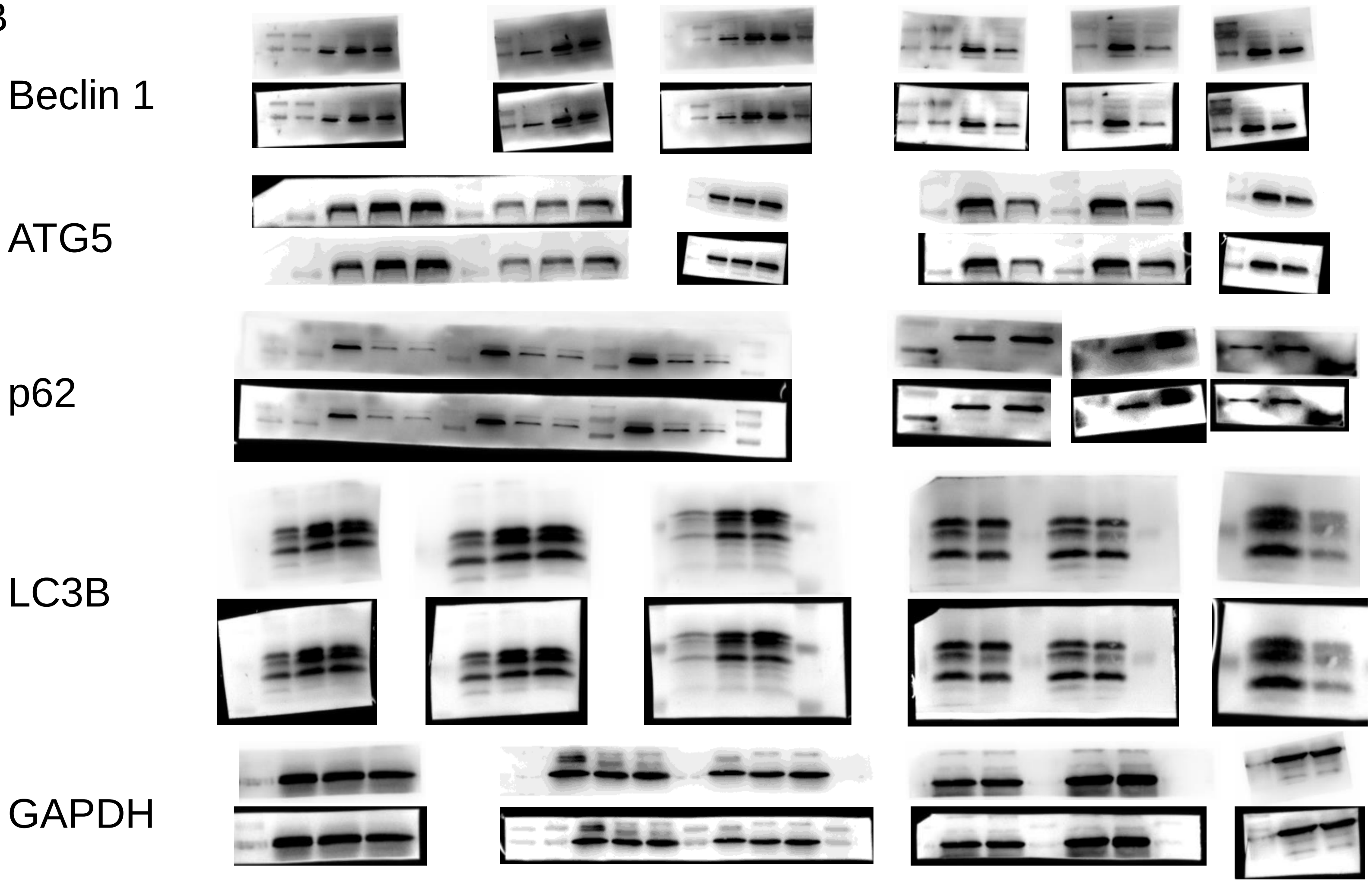

Figure 3

H

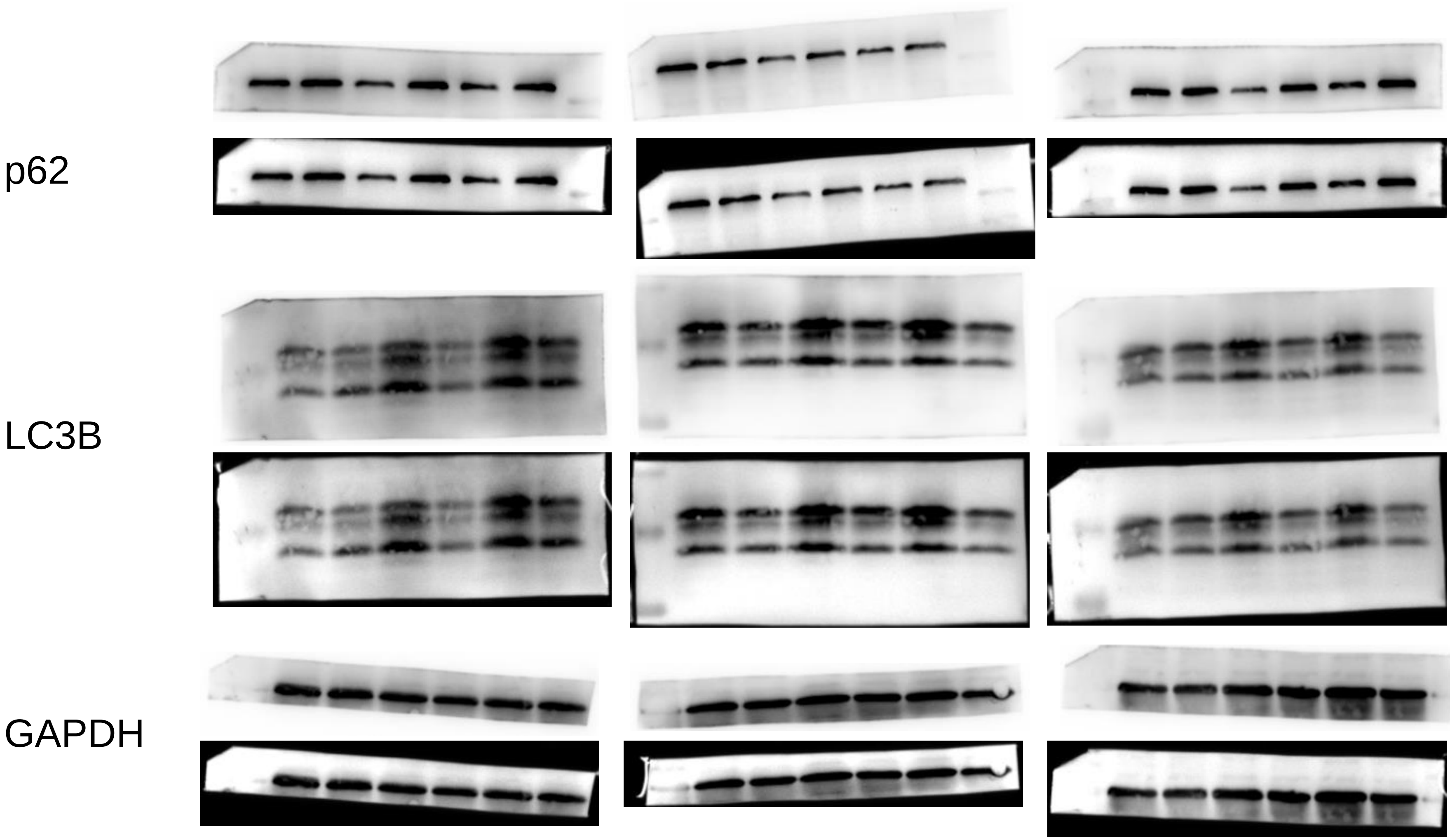

Figure 4

B

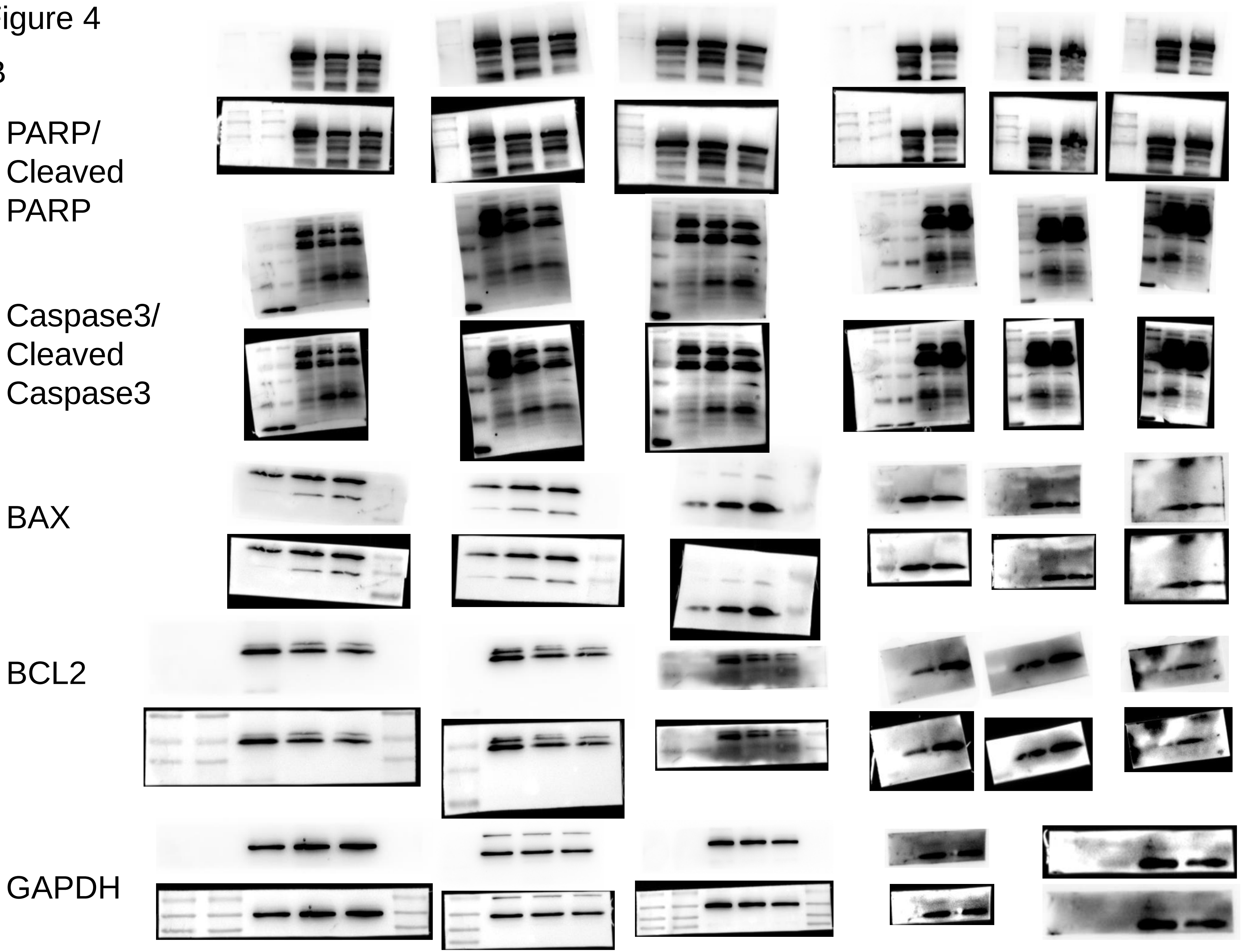
PARP/
Cleaved
PARP
Caspase3/
Cleaved
Caspase3
BAX
BCL2
GAPDH

Figure 4

I

PARP/
Cleaved
PARP

Caspase3/
Cleaved
Caspase3

GAPDH

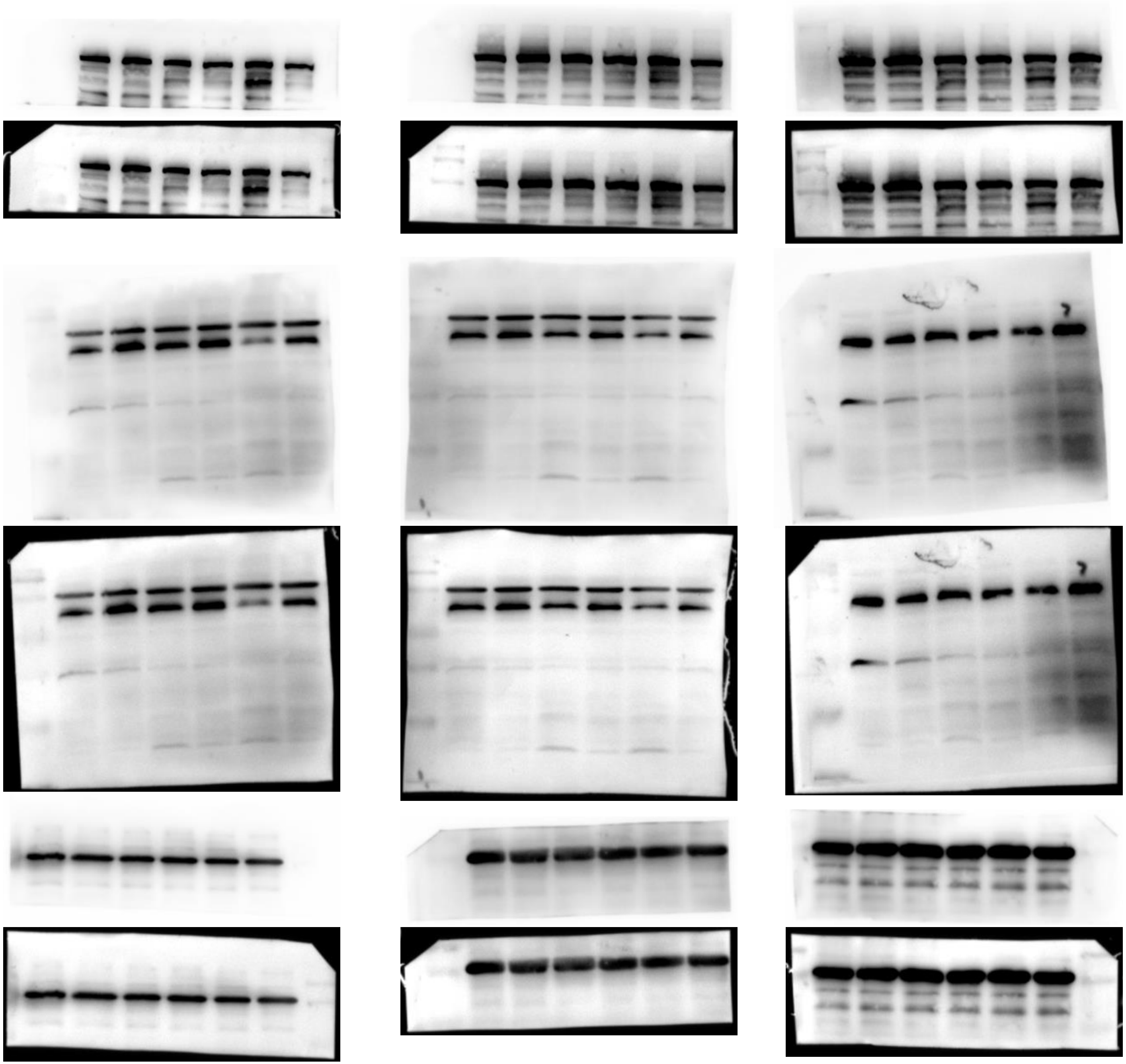

Figure 5

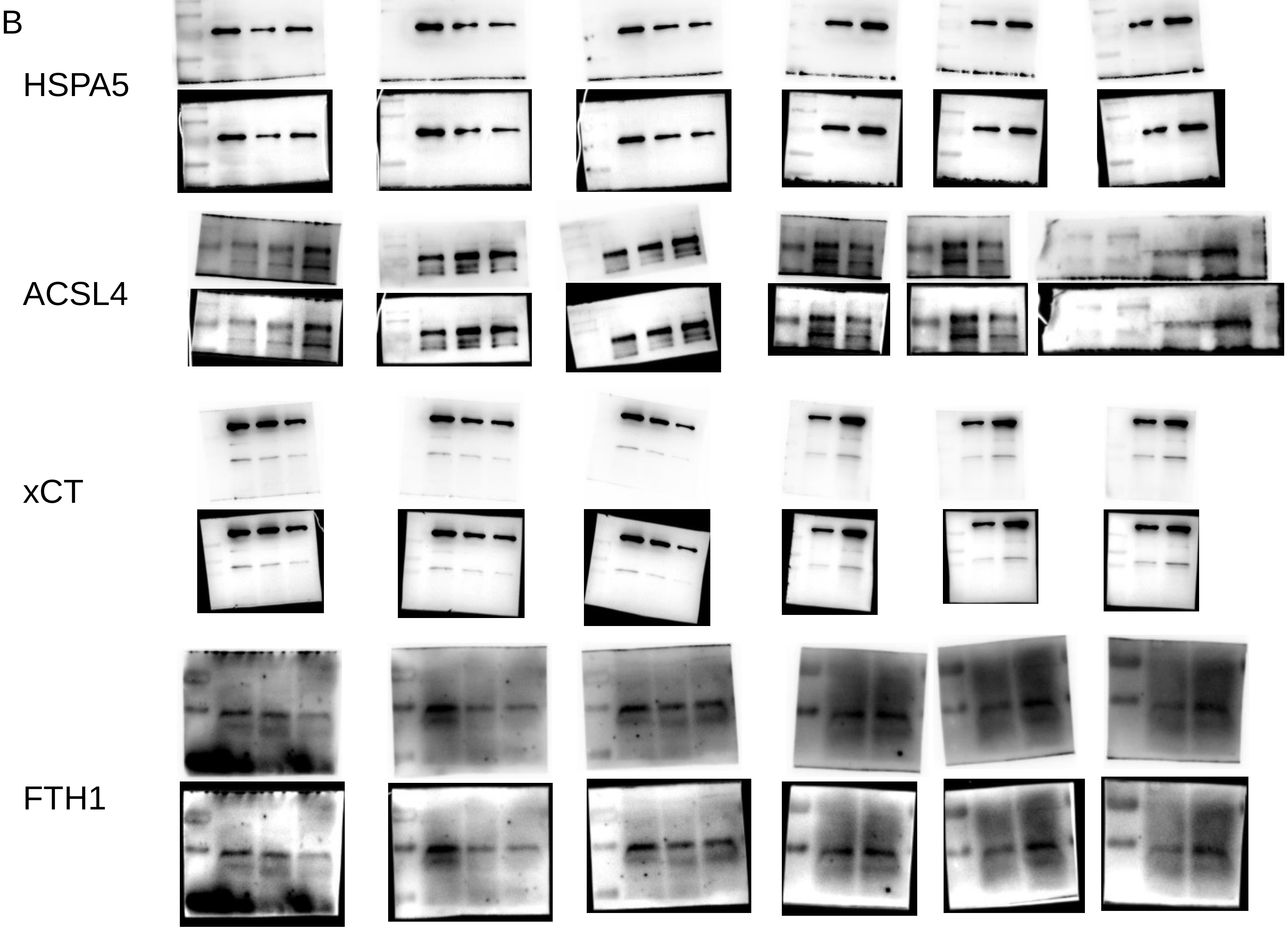

Figure 5

B

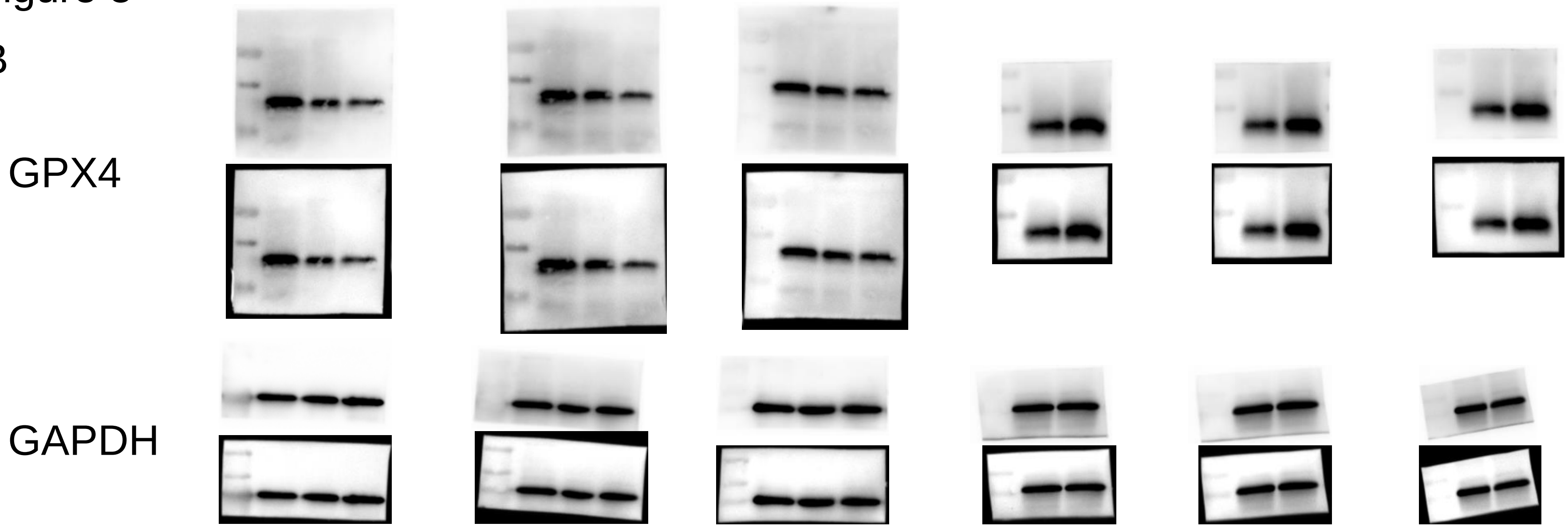

Figure 5

K

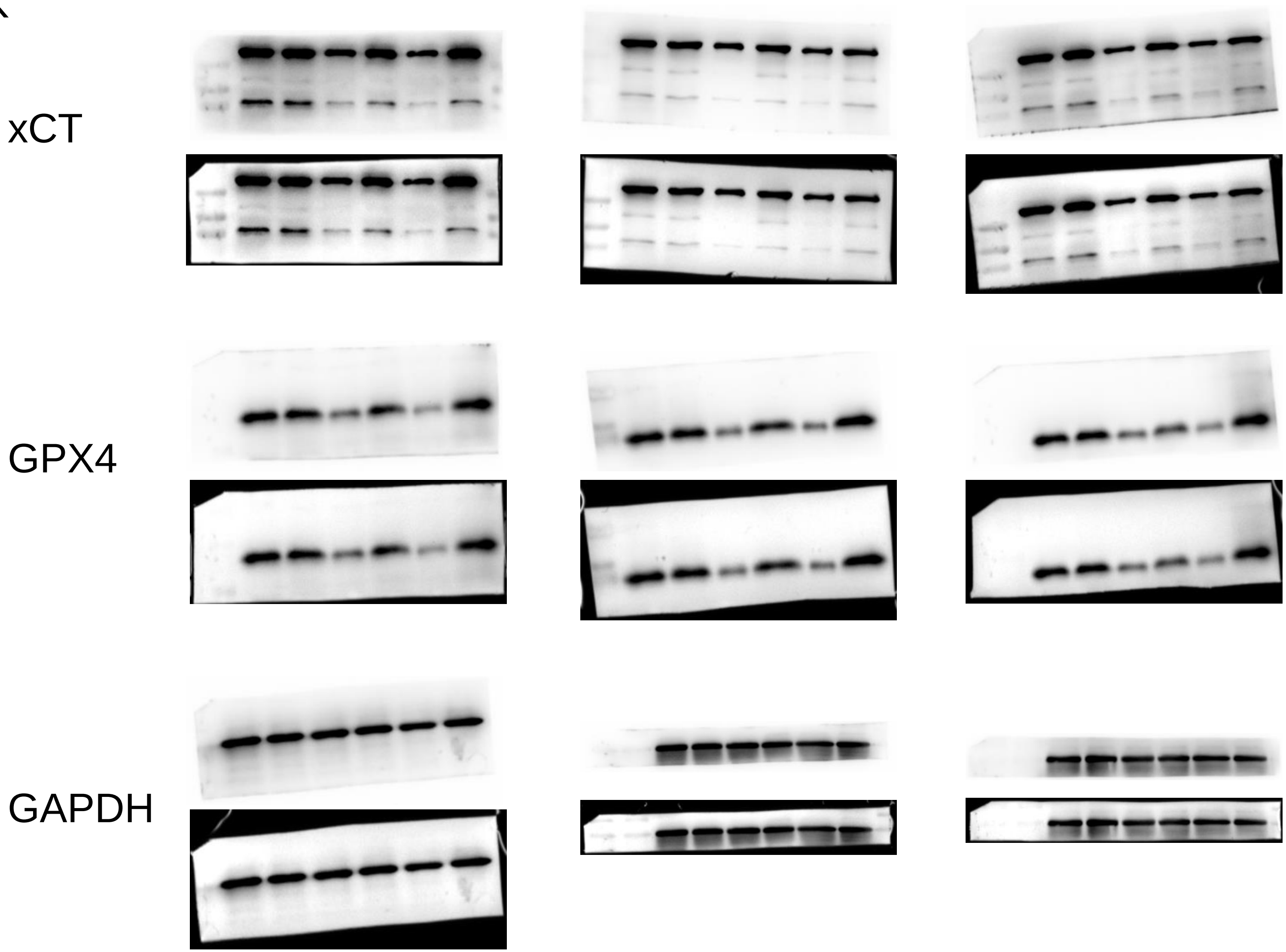

Figure 6

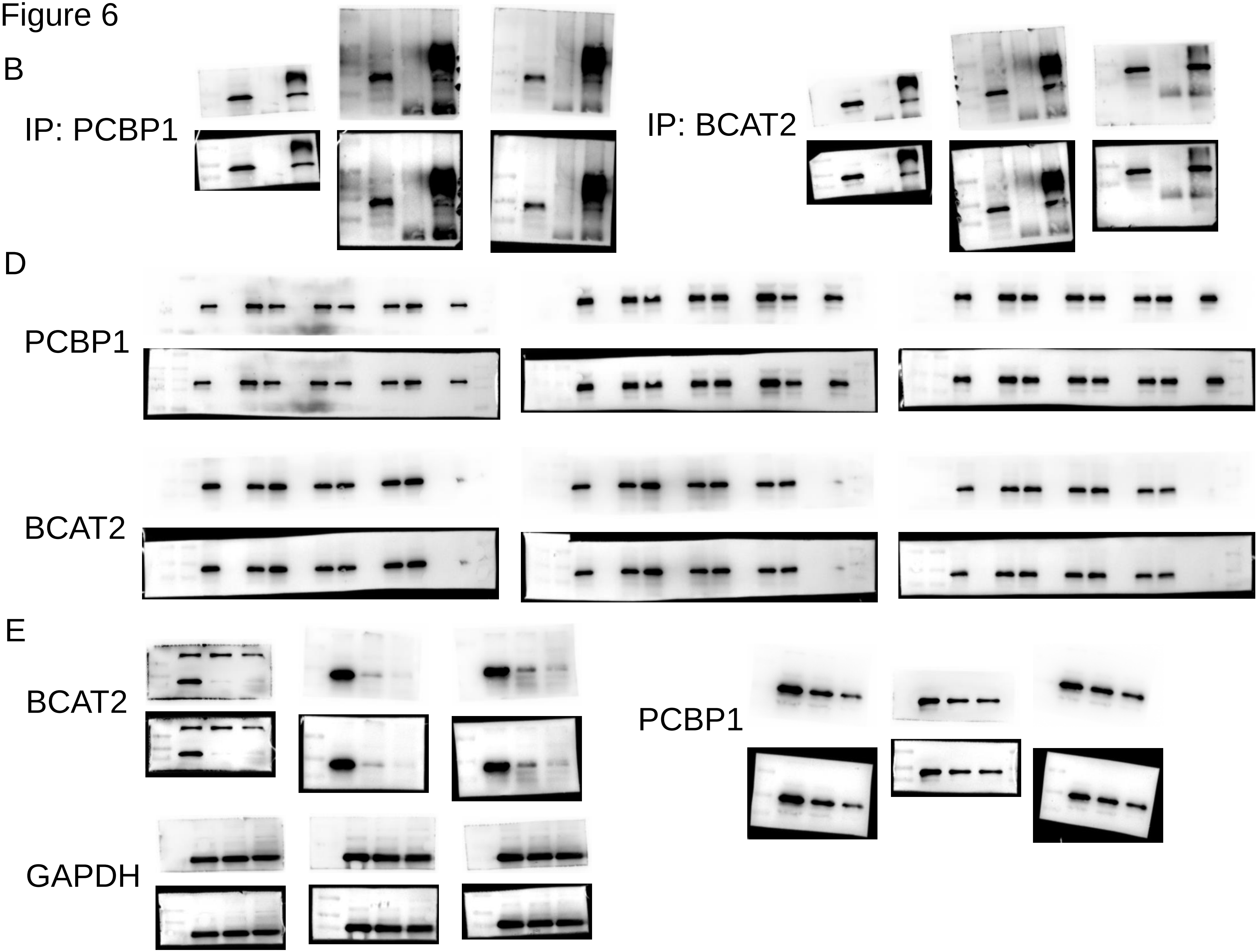

Figure 6

F

PCBP1

GAPDH

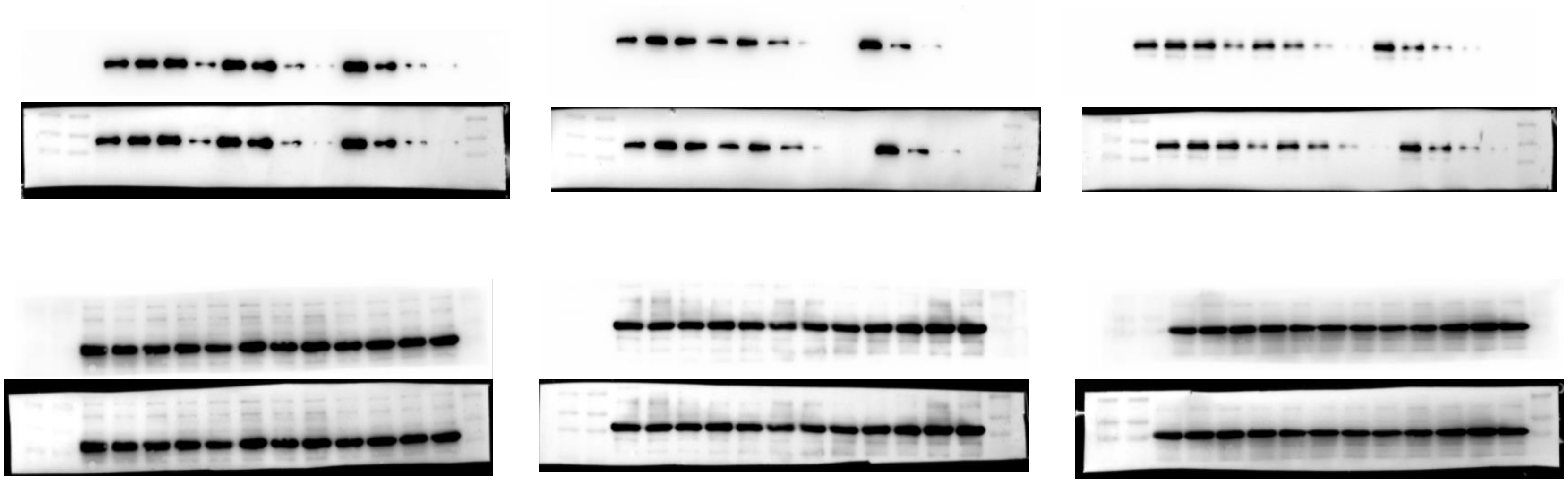

G

Ubiquitylation

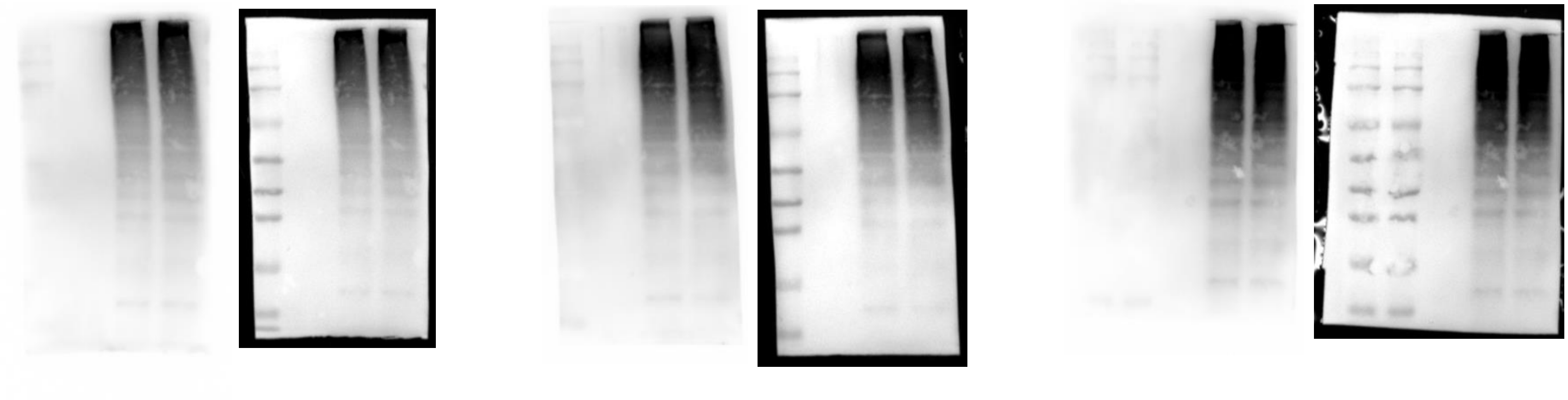

Figure 7

D

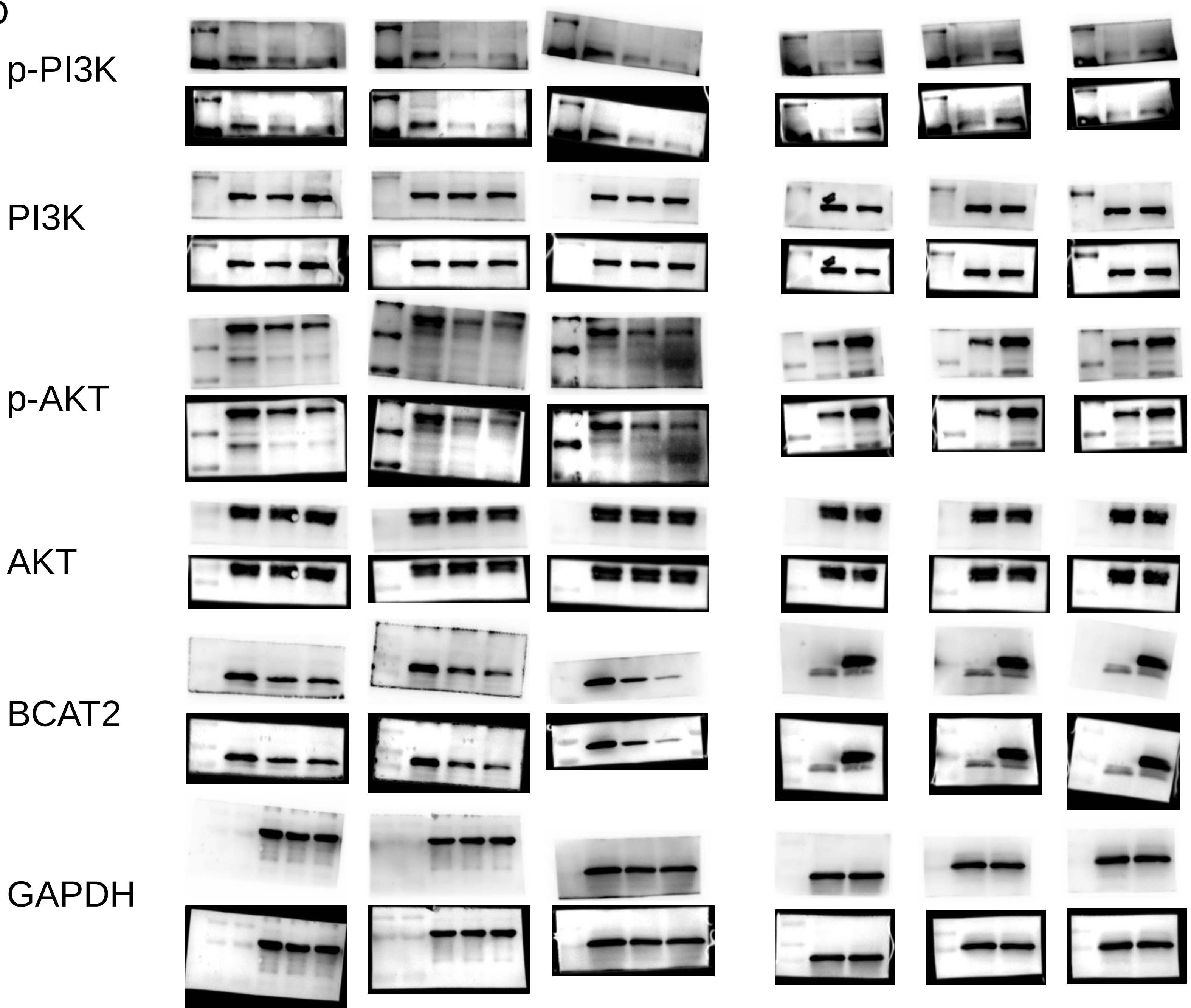

Figure 7

E

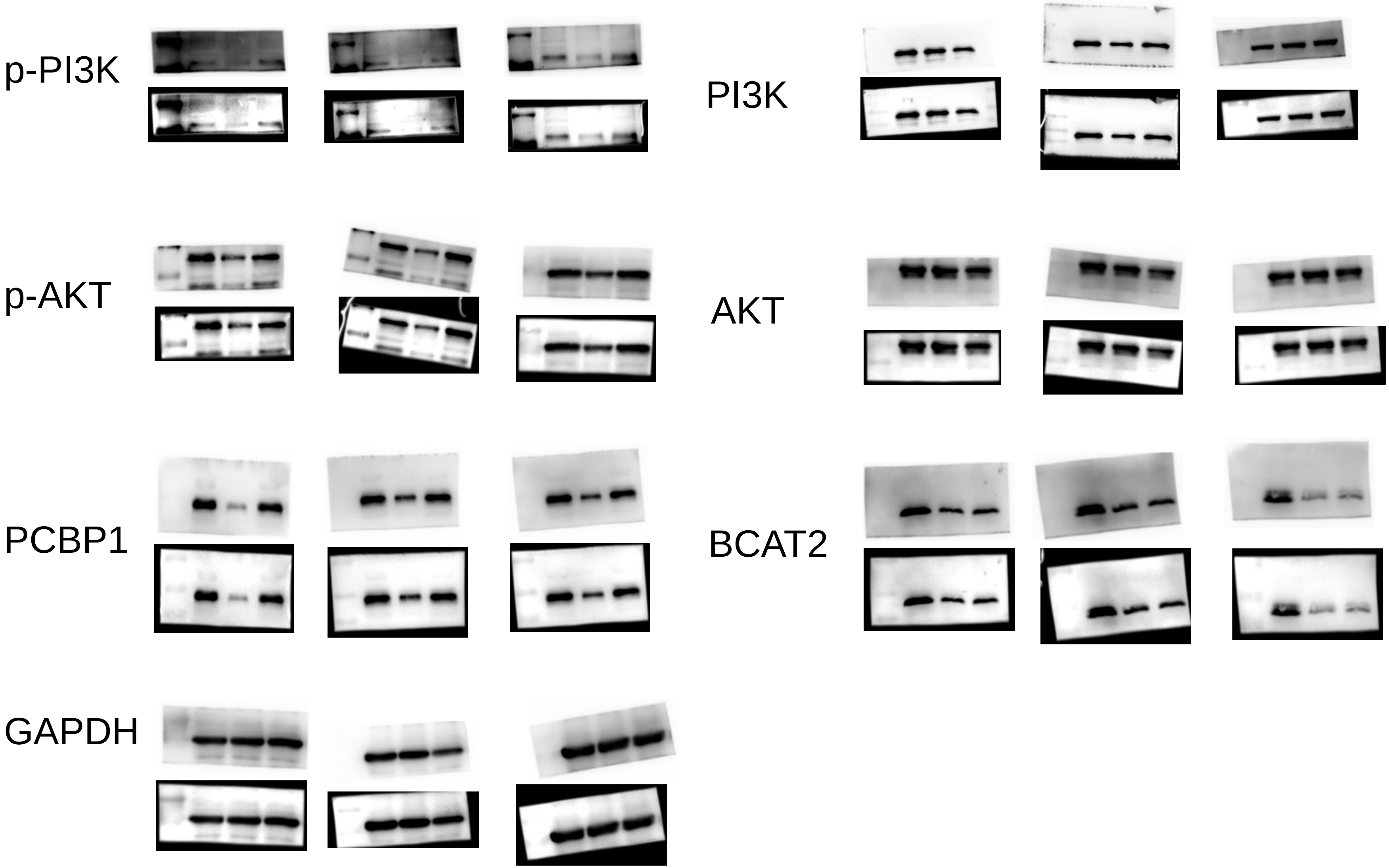

Figure S7

B

PCBP1

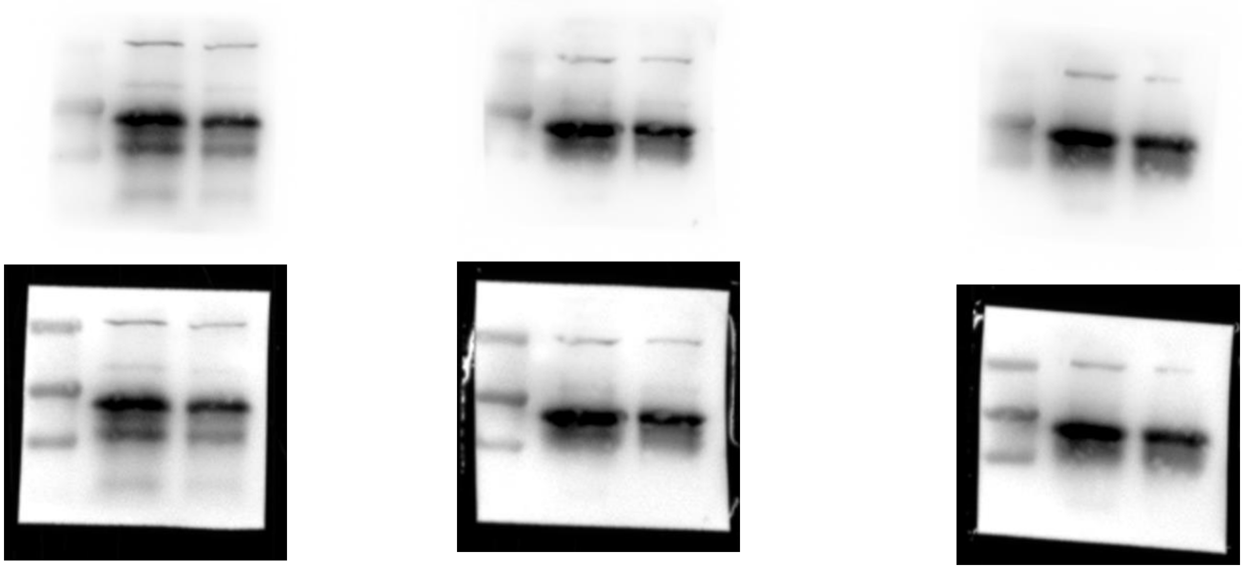

GAPDH

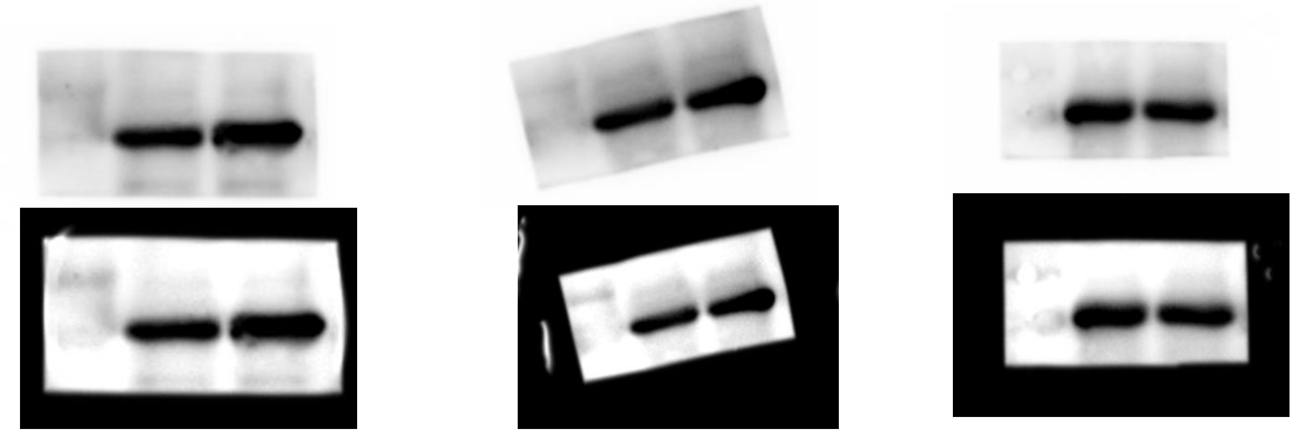

Figure S7

C

PCBP1

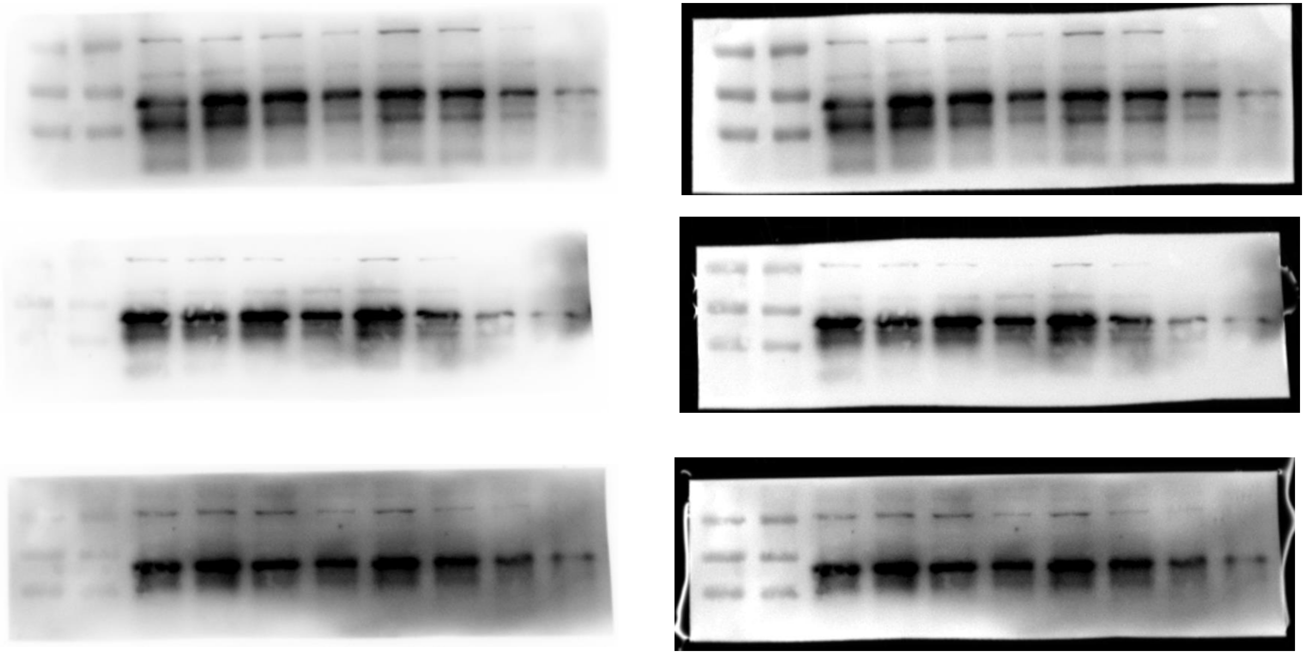

GAPDH

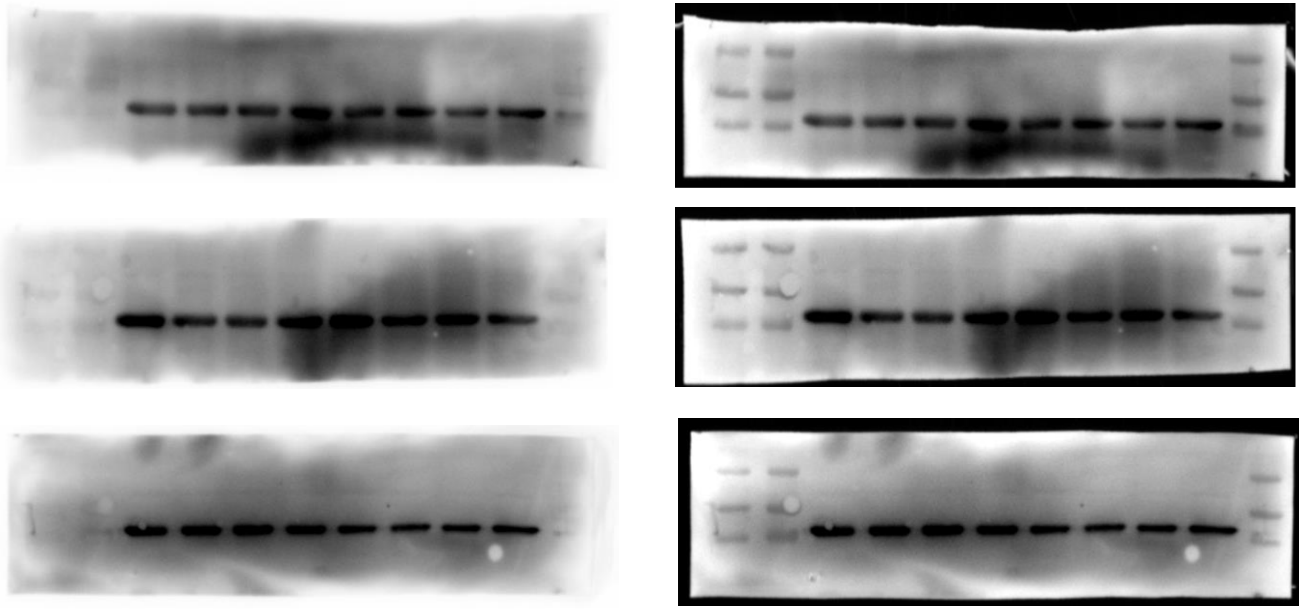

Supplement: Supplementary file 1 — Supplementary original blots [file 41419_2025_7559_MOESM1_ESM.pdf]
