# Supplementary material for: BCAT2 binding to PCBP1 regulates the PI3K/AKT signaling pathway to inhibit autophagy-related apoptosis and ferroptosis in prostate cancer
Source: Cell Death Dis. 2025 Apr 24;16(1):337. doi: 10.1038/s41419-025-07559-3 (PMC12022009; doi:10.1038/s41419-025-07559-3)
Supplement: Supplementary file 2 — Supplementary information [file 41419_2025_7559_MOESM2_ESM.docx]

Supporting Information

**Table S1: Primary antibodies list**

| Antibodies | Manufacturer | Catalog# |
| --- | --- | --- |
| GAPDH | Abcam | ab9485 |
| BCAT2 | Abclonal  Cell Signaling Technology | A23793  79764 |
| BCAT1 | ProteinTech | 13640-1-AP |
| BCKDHA | ProteinTech | 30028-1-AP |
| BCKDHB | ProteinTech | 13685-1-AP |
| Beclin 1 | Abclonal | A21191 |
| ATG5 | Abclonal | A11427 |
| p62 | Abclonal | A19700 |
| LC3B | Abclonal | A19665 |
| PARP | Cell Signaling Technology | 9532T |
| Caspase 3 | Cell Signaling Technology | 9664T |
| BAX | Abcam | ab32503 |
| BCL2 | Abcam | ab182858 |
| HSPA5 | Abclonal | A23453 |
| ACSL4 | Abclonal | A20414 |
| xCT | Abclonal | A2413 |
| FTH1 | Abclonal | [A19544](https://abclonal.com.cn/catalog/A19544) |
| GPX4 | Abmart | [T56959](http://www.ab-mart.com.cn/page.aspx?node=%2077%20&id=%202627) |
| PCBP1 | [Santa Cruz Biotechnology](https://www.scbt.com/zh/home) | sc-393076 |
| Flag | Abclonal | AE092 |
| Myc | Abclonal | AE070 |
| PI3K | Abmart | [T40115](http://www.ab-mart.com.cn/page.aspx?node=%2077%20&id=%2049665) |
| p-PI3K | Abcam | ab278545 |
| AKT | Abmart | [T55561](http://www.ab-mart.com.cn/page.aspx?node=%2077%20&id=%201801) |
| p-AKT | Abclonal | AP1453 |
| Ubiquitin | Abclonal | A19686 |

**Table S2: The sequence of the shRNAs**

| ShRNA |  | Sequence (5'- 3') | |
| --- | --- | --- | --- |
| shNC  shBCAT2-1 | Forward  Reverse  Forward | | GATCTGTTCTCCGAACGTGTCACGTTTCAAGAGAACGTGACACGTTCGGAGAATTTTTTC  AATTGAAAAAATTCTCCGAACGTGTCACGTTCTCTTGAAACGTGACACGTTCGGAGAACA  gatccAGGGCATGAAGGCGTTCAAAGCTCGAGCTTTGAACGCCTTCATGCCCTTTTTTT  aattAAAAAAAGGGCATGAAGGCGTTCAAAGCTCGAGCTTTGAACGCCTTCATGCCCTg |
|  | Reverse | |  |
| shBCAT2-2 | Forward  Reverse | | gatccGTGGGAACCATGAACATCTTTGCTCGAGCAAAGATGTTCATGGTTCCCATTTTTT  aattAAAAAATGGGAACCATGAACATCTTTGCTCGAGCAAAGATGTTCATGGTTCCCACg |
| shBCAT2-3 | Forward | | gatccGTGCACCGAATCCTGTACAAAGCTCGAGCTTTGTACAGGATTCGGTGCATTTTTT  aattAAAAAATGCACCGAATCCTGTACAAAGCTCGAGCTTTGTACAGGATTCGGTGCACg |
|  | Reverse | |  |

**Table S3: The univariate and multivariate Cox regression analyses**

| Clinlcopathological feature | Hazard ration  (95% CI) | *p* |
| --- | --- | --- |
| **Univariate analysis** |  |  |
| PSA | 1.386 (0.970-1.980) | 0.073 |
| T stage | 1.854 (1.287-2.673) | < 0.001 |
| N stage | 1.675 (1.068-2.625) | 0.024 |
| Gleason score  BCAT2 expression | 1.481 (1.044-2.100)  3.157 (2.136-4.665) | 0.027  < 0.001 |
| **Multivariate analysis** |  |  |
| PSA | 1.167 (0.796-1.711) | 0.429 |
| T stage | 1.220 (0.789-1.888) | 0.371 |
| N stage | 1.520 (0.930-2.482) | 0.095 |
| Gleason score  BCAT2 expression | 0.960 (0.639-1.443)  2.923 (1924-4.440) | 0.846  < 0.001 |


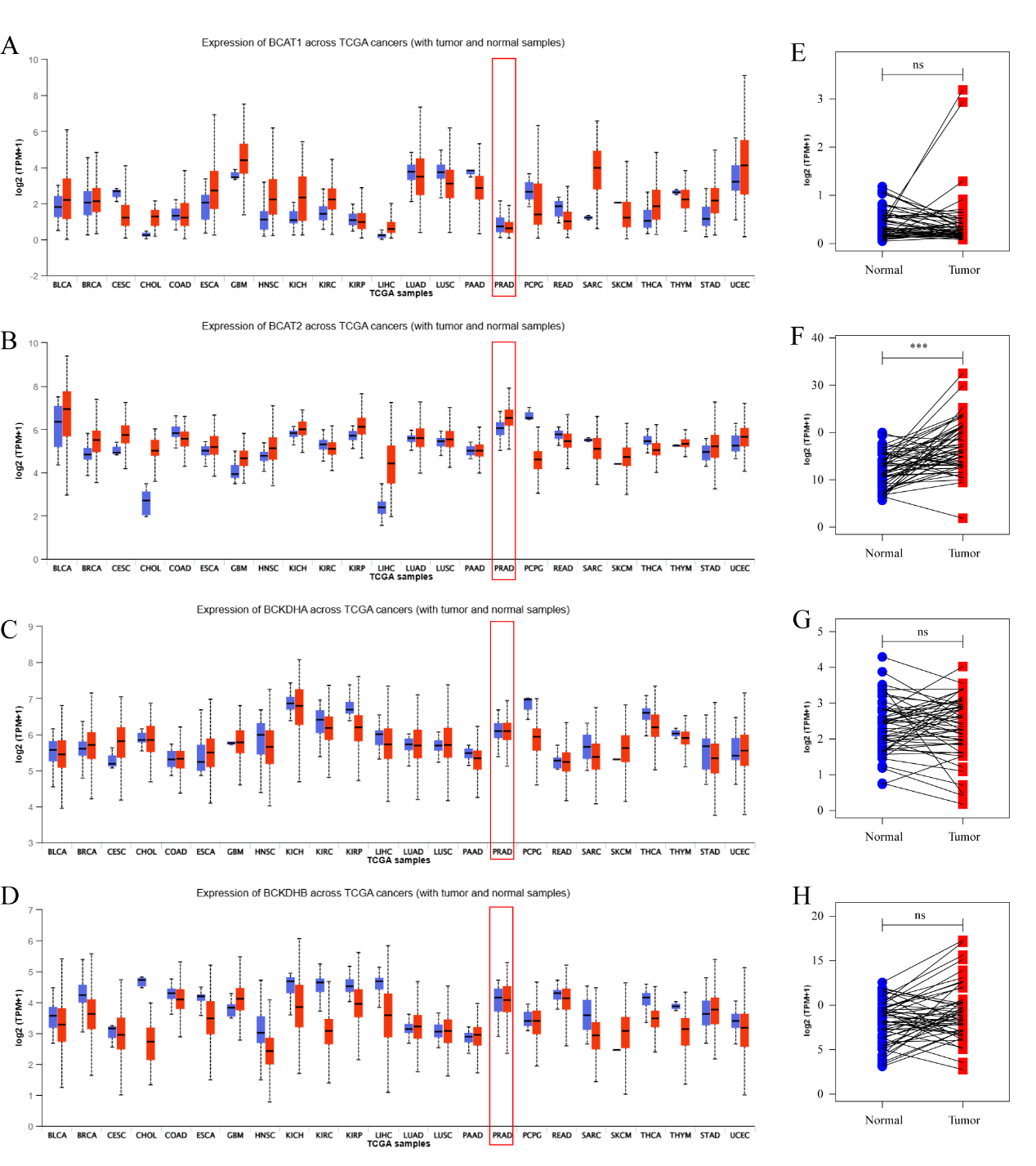


**Fig. S1** (A) BCAT1, (B) BCAT2, (C) BCKDHA, and (D) BCKDHB expression in PCa in TCGA database. (E) BCAT1, (F) BCAT2, (G) BCKDHA, and (H) BCKDHB expression in paired PCa and paracancerous tissues in TCGA database.


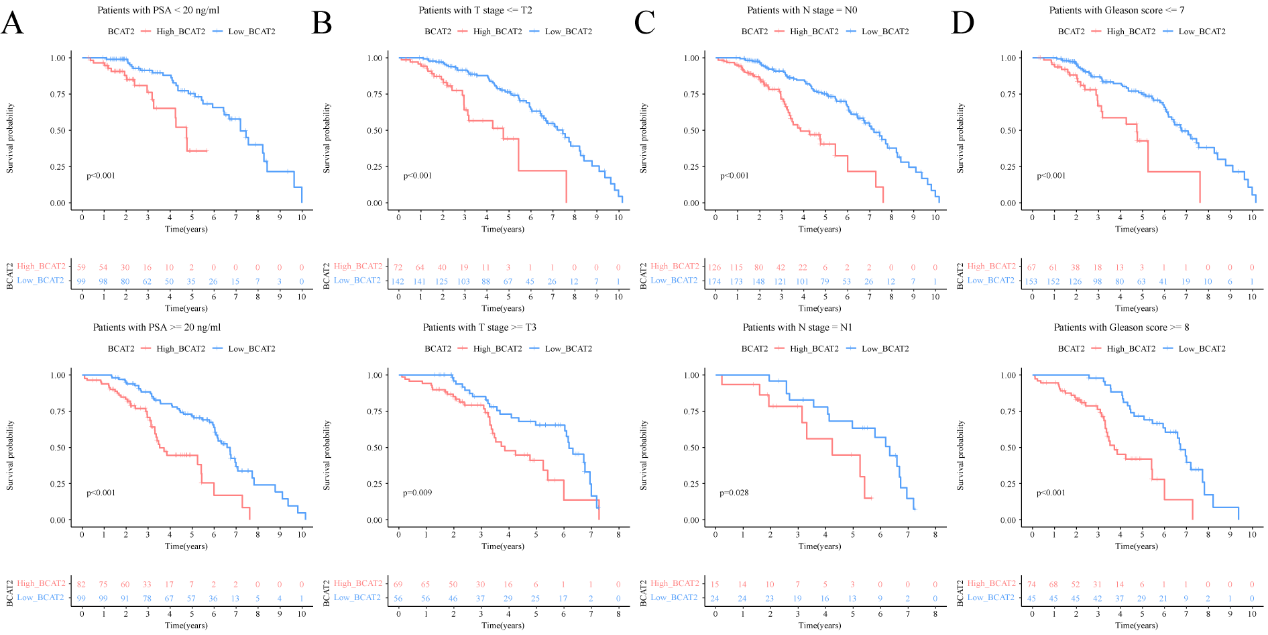


**Fig. S2** KM curves of BFFS for PCa patient with different (A) PSA, (B) T stage, (C) N stage, and (D) Gleason score.


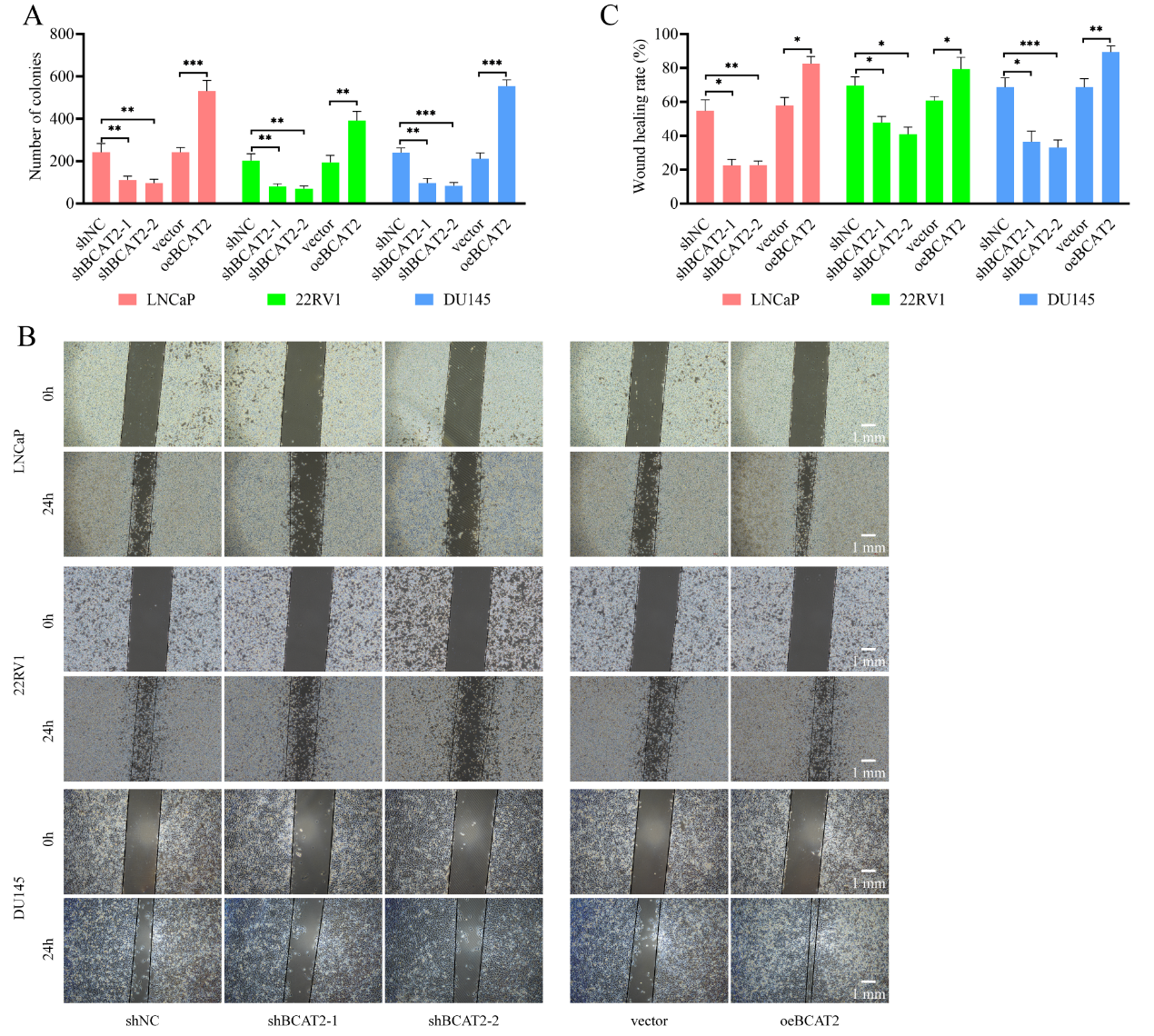


**Fig. S3** (A) Statistical analysis of clone formation assay investigating the effects of BCAT2 on cell proliferative. (B, C) BCAT2 knockdown attenuated cell migration, whereas BCAT2 overexpression exerted the opposite effect.


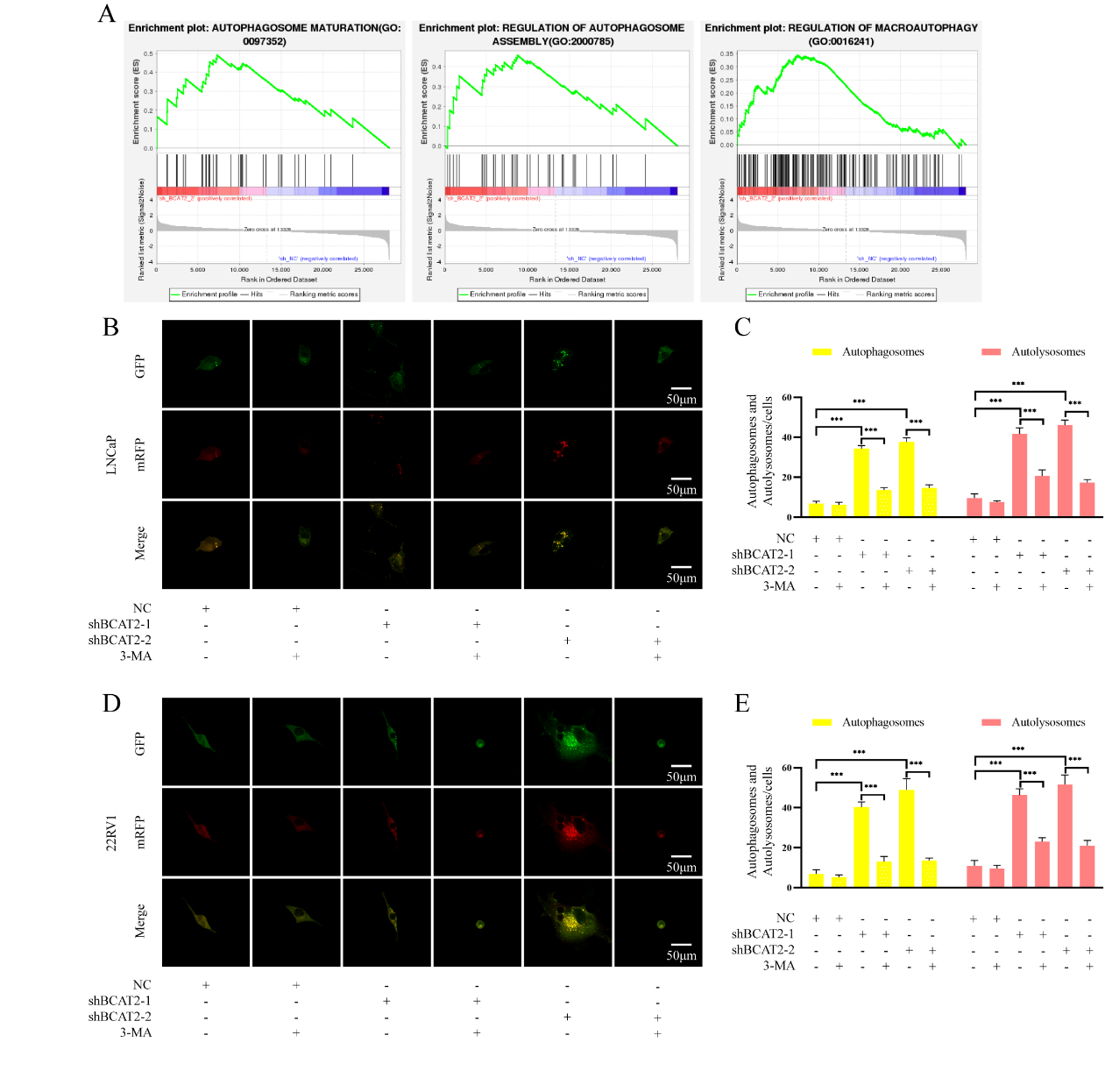


**Fig. S4** (A) GSEA analysis of some autophagy related pathways. (B, C) LNCaP and (D, E) 22RV1 shNC or shBCAT2-2 cells transfected with mCherry-GFP-LC3B plasmid were treated with or without 5 mM 3-MA for 24 h. The average number of red dots (autolysosomes) and yellow dots (autophagosomes) was quantified.


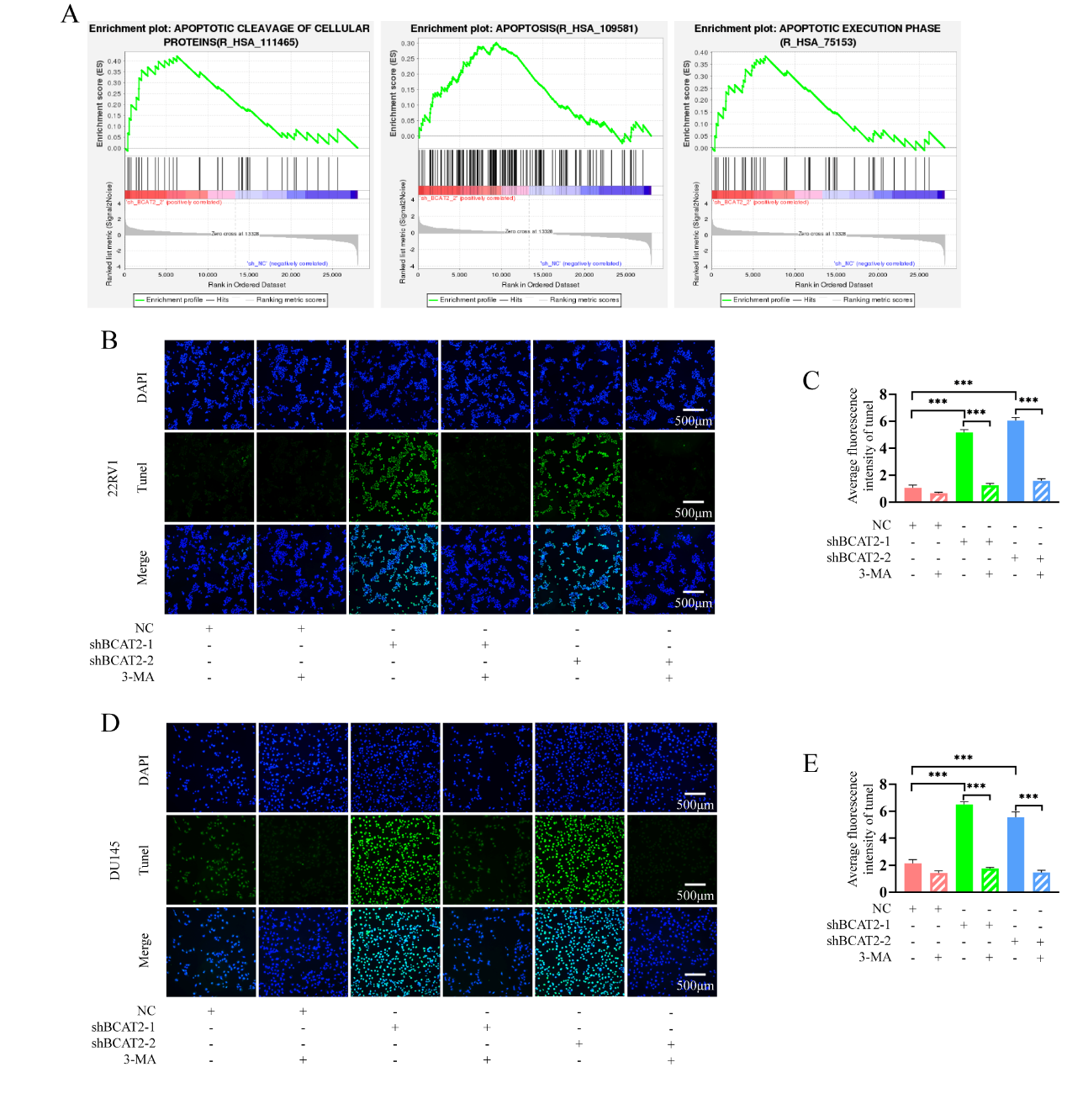


**Fig. S5** (A) GSEA analysis of some apoptosis related pathways. (B, C) 22RV1 and (D, E) DU145 shNC or shBCAT2 cells, treated with 5 mM 3-MA for 24 h, stained with TUNEL.


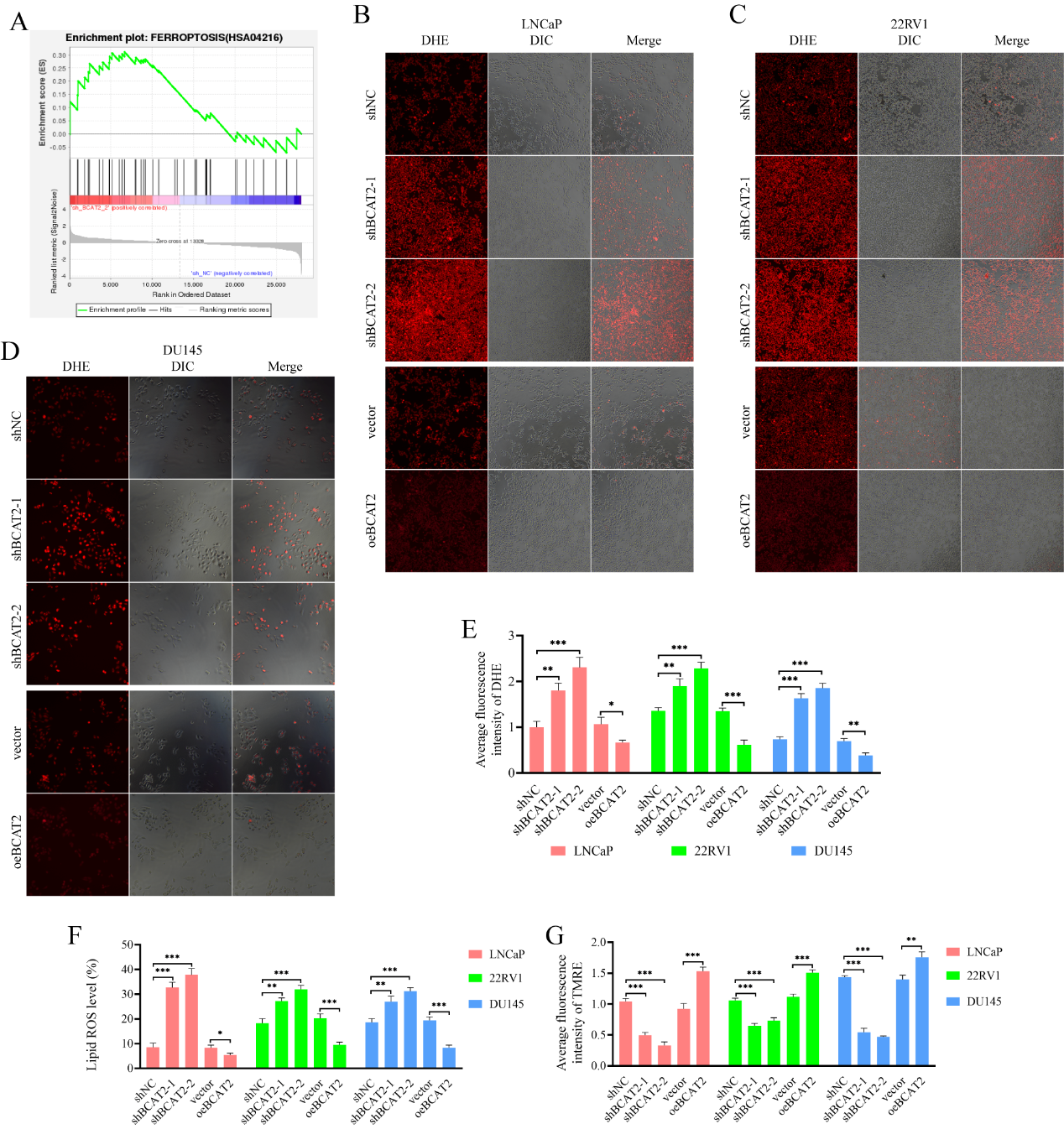


**Fig. S6** (A) GSEA analysis of some ferroptosis pathway. (B-E) DHE levels in were increased with the knockdown of BCAT2, whereas decreased upon BCAT2 overexpression. (F) Statistical analysis of the lipid ROS level (C11-BODIPY^®^ 581/591) of PCa cells. (G) Statistical analysis of TMRE staining in PCa cells.


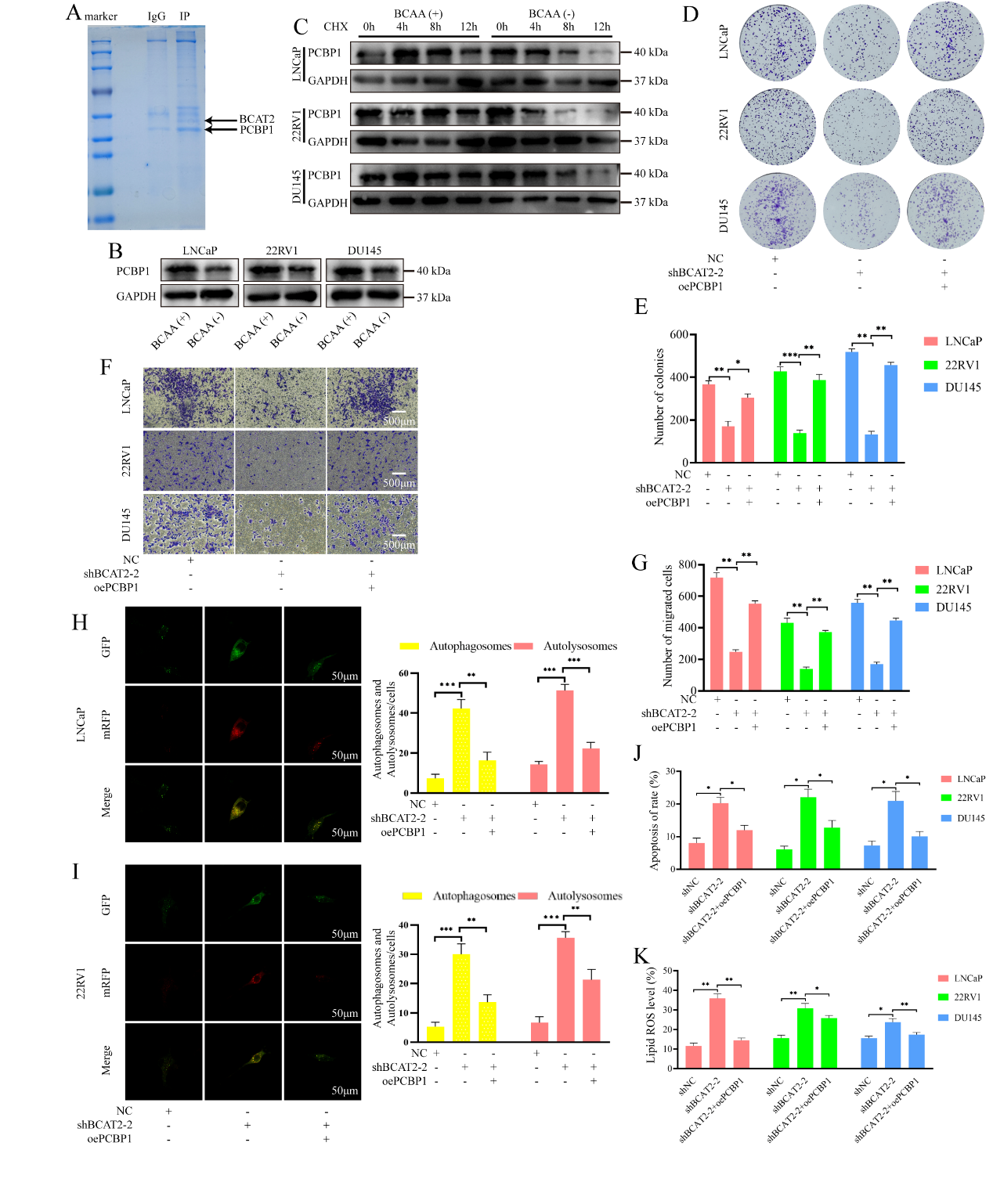


**Fig. S7** (A) The gel stained with coomassie brilliant blue to show separated proteins. (B) WB showed that PCBP1 protein level in PCa cells was inhibited after cultured with BCAA-free medium for 48h. (C) After cultured with BCAA-free medium, the PCBP1 protein level was detected. The cells were then treated with cycloheximide for specified time. (D, E). Clonal formation assay of PCa cells, transfected with shBCAT2 and/or oePCBP1. (F, G). Transwell assay of PCa cells, transfected with shBCAT2 and/or oePCBP1. (H) LNCaP and (I) 22RV1 cells transfected with mCherry-GFP-LC3B plasmid, also transfected with shBCAT2 and/or oePCBP1. (J) Statistical analysis of PCa cells, transfected with shBCAT2 and/or oePCBP1. (K) Statistical analysis of the lipid ROS level of PCa cells, transfected with shBCAT2 and/or oePCBP1.


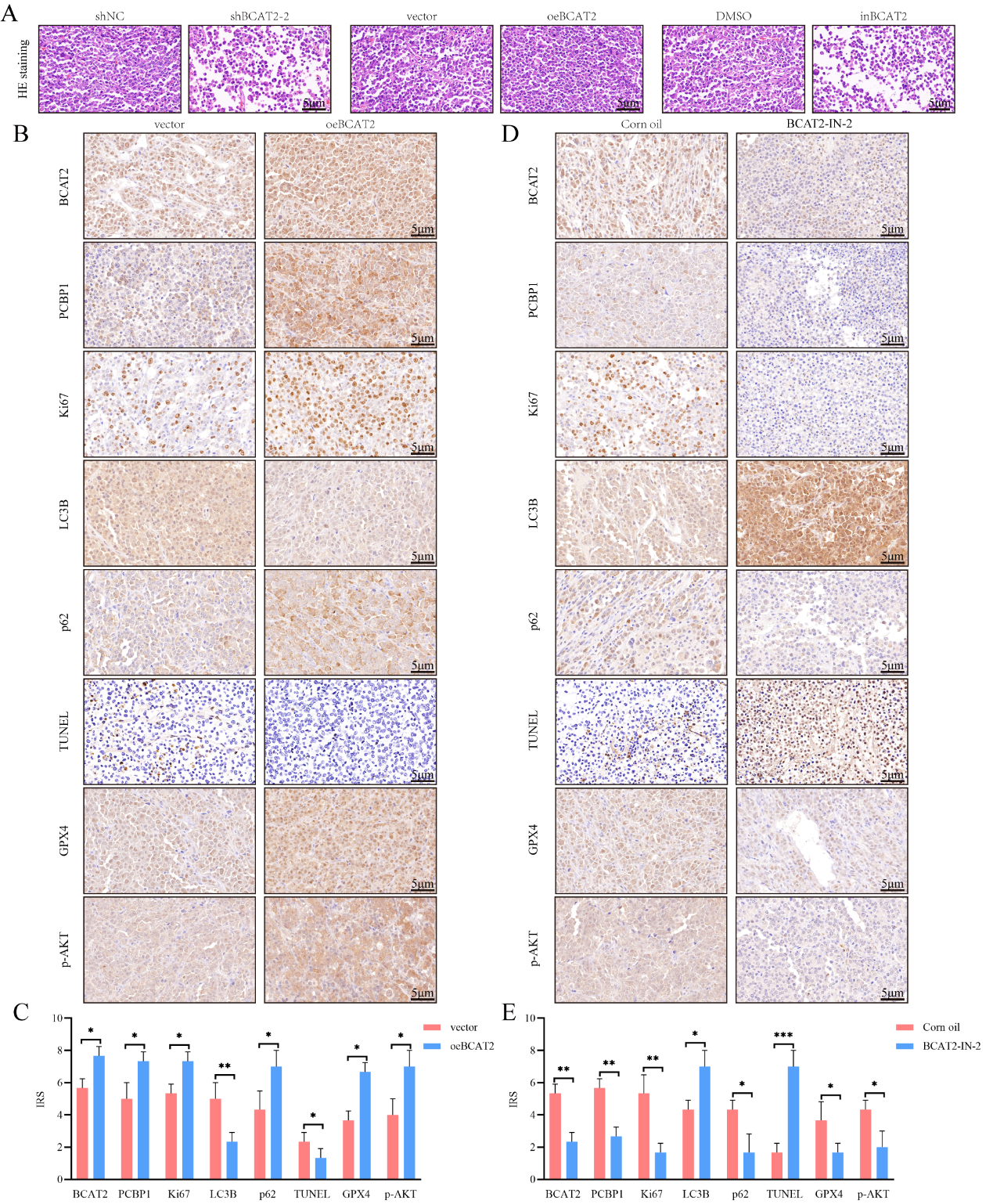


**Fig. S8** (A) Hematoxylin-eosin staining of tumor in different groups of mice. (B, C) Immunohistochemical staining of specified indicator in the BCAT2 overexpression group. (D, E) Immunohistochemical staining of specified indicator in the BCAT2-IN-2 or corn oil intragastric group.
